# Effects of N-Glycosylation on the Structure, Function, and Stability of a Plant-Made Fc-Fusion Anthrax Decoy Protein

**DOI:** 10.3389/fpls.2019.00768

**Published:** 2019-06-28

**Authors:** Yongao Xiong, Kalimuthu Karuppanan, Austen Bernardi, Qiongyu Li, Vally Kommineni, Abhaya M. Dandekar, Carlito B. Lebrilla, Roland Faller, Karen A. McDonald, Somen Nandi

**Affiliations:** ^1^ Department of Chemical Engineering, University of California, Davis, Davis, CA, United States; ^2^ Department of Chemistry, University of California, Davis, Davis, CA, United States; ^3^ iBio CDMO LLC, Bryan, TX, United States; ^4^ Department of Plant Sciences, University of California, Davis, Davis, CA, United States; ^5^ Department of Biochemistry and Molecular Medicine, University of California, Davis, Davis, CA, United States; ^6^ Global HealthShare Initiative, University of California, Davis, Davis, CA, United States

**Keywords:** anthrax decoy protein, N-glycosylation, molecular simulation, protein stability, kinetics of protein binding

## Abstract

Protein N-glycosylation is an important post-translational modification and has influences on a variety of biological processes at the cellular and molecular level, making glycosylation a major study aspect for glycoprotein-based therapeutics. To achieve a comprehensive understanding on how N-glycosylation impacts protein properties, an Fc-fusion anthrax decoy protein, *viz* rCMG2-Fc, was expressed in *Nicotiana benthamiana* plant with three types of N-glycosylation profiles. Three variants were produced by targeting protein to plant apoplast (APO), endoplasmic reticulum (ER) or removing the N-glycosylation site by a point mutation (Agly). Both the APO and ER variants had a complex-type N-glycan (GnGnXF) as their predominant glycans. In addition, ER variant had a higher concentration of mannose-type N-glycans (50%). The decoy protein binds to the protective antigen (PA) of anthrax through its CMG2 domain and inhibits toxin endocytosis. The protein expression, sequence, N-glycosylation profile, binding kinetics to PA, toxin neutralization efficiency, and thermostability were determined experimentally. In parallel, we performed molecular dynamics (MD) simulations of the predominant full-length rCMG2-Fc glycoform for each of the three N-glycosylation profiles to understand the effects of glycosylation at the molecular level. The MAN8 glycoform from the ER variant was additionally simulated to resolve differences between the APO and ER variants. Glycosylation showed strong stabilizing effects on rCMG2-Fc during *in planta* accumulation, evidenced by the over 2-fold higher expression and less protein degradation observed for glycosylated variants compared to the Agly variant. Protein function was confirmed by toxin neutralization assay (TNA), with effective concentration (EC_50_) rankings from low to high of 67.6 ng/ml (APO), 83.15 ng/ml (Agly), and 128.9 ng/ml (ER). The binding kinetics between rCMG2-Fc and PA were measured with bio-layer interferometry (BLI), giving sub-nanomolar affinities regardless of protein glycosylation and temperatures (25 and 37°C). The protein thermostability was examined utilizing the PA binding ELISA to provide information on EC_50_ differences. The fraction of functional ER variant decayed after overnight incubation at 37°C, and no significant change was observed for APO or Agly variants. In MD simulations, the MAN8 glycoform exhibits quantitatively higher distance between the CMG2 and Fc domains, as well as higher hydrophobic solvent accessible surface areas (SASA), indicating a possibly higher aggregation tendency of the ER variant. This study highlights the impacts of N-glycosylation on protein properties and provides insight into the effects of glycosylation on protein molecular dynamics.

## Introduction

Anthrax is a severe infectious disease caused by *Bacillus anthracis*. The spores can be produced easily and released in air as a biological weapon, leading to a fatality rate of 86–89% (Kamal et al., 2011). *Bacillus anthracis* secrets anthrax toxin, which is composed of a cell-binding protein, namely protective antigen (PA), and two enzymatic proteins called lethal factor (LF) and edema factor (EF). The cellular toxicity starts with the binding of PA to anthrax toxin receptors, after which the bound PA is cleaved by a furin family protease, leaving a 63 kDa fragment bound to the receptors (Wigelsworth et al., 2004). The receptor-PA complex then self-assembles into a heptamer (PA)_7_, allowing binding of LF and EF, which is then internalized to the cytosol through endocytosis, causing disruption to normal cellular physiology (Wigelsworth et al., 2004). Antitoxins based on receptor-decoy binding show promising advantages over an antibody-based strategy since it is difficult to engineer toxins to escape the inhibitory effect of the decoy without compromising binding to its cellular receptor. By making the extracellular domain of the main anthrax toxin receptor Capillary Morphogenesis Gene 2 protein recombinantly (rCMG2), that can be used as a prophylaxis or post-exposure treatment, to neutralize anthrax toxins in blood, preventing cell infection. Additionally, fusing an Fc domain to rCMG2 increases the serum half-life through interaction with the salvage neonatal Fc-receptor (Roopenian and Akilesh, 2007) and lowers renal clearance rate (Knauf et al., 1988). These factors make rCMG2-Fc a promising anthrax decoy protein, which retains the high binding affinity to the PA along with a longer blood circulatory half-life than rCMG2 (Wycoff et al., 2011; Xi et al., 2014; Karuppanan et al., 2017). We used a plant-based expression system for protein expression due to its rapid production rate and inherent scalability, which is critical for providing rapid response under emergency conditions. Moreover, plants rarely carry animal pathogens and are capable of post-translational modification, making them an appealing alternative to traditional protein expression systems such as mammalian cell culture or microbial fermentation (Chen and Davis, 2016).

N-glycosylation can affect protein folding, structural integrity, and function (Mimura et al., 2000; Krapp et al., 2003), which makes it an important design consideration for glycoprotein-based therapeutics. In some cases, proteins with proper glycosylation exhibit optimal efficacy. For example, Fc glycosylation is required to elicit effector functions of human IgG1 (Hristodorov et al., 2013). Thus, it should be preserved when immune defense is desired, for instance, when expressing antitumor mAbs (Strome et al., 2007). On the other hand, for drugs that treat chronic conditions, the absence of glycosylation is desired to avoid effector functions and associated inflammatory responses. Another important consideration is that glycosylated proteins are less susceptible to proteases, such as pepsin, compared with aglycosylated counterparts (Niu et al., 2016), which should be considered to maximize protein yield.

Although the impacts of protein N-glycosylation have been studied, typically only one or two aspects were studied at a time, and these studies were done on antibodies (Raju and Scallon, 2006; Kayser et al., 2011; Zheng et al., 2011). This study provides a comprehensive approach utilizing a combination of experimental and computational techniques to evaluate the effects of N-glycosylation on rCMG2-Fc fusion protein properties. In this study, the protein expression, toxin neutralization efficacy, binding kinetics, thermostability, and structural configuration were studied experimentally and compared among three rCMG2-Fc glycoform variants. In addition, we employ atomistic molecular dynamics (MD) simulation to understand the structure and dynamics of the predominant glycoform of the APO, ER, and Agly variants. Atomistic MD simulations are well-suited for the study of biomolecular systems, providing full accessibility to virtual, high-resolution, time-ordered, atomic trajectories (Dror et al., 2012). MD simulations have been used to study many different biological systems, including lipid membranes, trans-membrane proteins, and other glycoproteins (Nury et al., 2010; Delemotte and Tarek, 2012; Bernardi et al., 2017). While fully atomistic protein simulation is a powerful tool to investigate structural and functional information, it is important to recognize the current limitations of the technique. In particular, protein folding is known to occur on the order of microseconds to seconds (Dill and MacCallum, 2012), while atomistic protein simulation is generally limited to hundreds of nanoseconds due to limited computing resources. This limitation generally prohibits the straightforward simulation of protein fold transitions. The length-scale of atomistic protein simulations is also computationally restricted, allowing only one rCMG2-Fc dimer to be simulated. Despite these limitations, this work shows MD simulation data is capable of providing insight into the effects of glycosylation on protein structure, and improving our understanding and interpretation of experimental observations. To the best of our knowledge, no study has been conducted on Fc-fusion protein considering that many experimental and molecular simulation factors. This study provides an integrated experimental and computational approach to evaluate Fc N-glycosylation impacts on rCMG2-Fc properties, and potentially serves as a guideline for general glycoprotein-based therapeutic design, especially for Fc-fusion proteins.

## Materials and Methods

### Gene Constructs

The codon optimized CMG2-Fc sequence includes the extracellular domain of CMG2 (amino acids 34–220, Genbank: AY233452), followed by two serine residues, the upper hinge of IgG2 (amino acids 99–105, Genbank: AJ250170.1), and Fc region of human IgG1 (amino acid 108–329, Genbank: AAC82527.1). The resulting sequence corresponds to the APO variant as described previously (Karuppanan et al., 2017). A SEKDEL C-terminal motif was included to make the ER variant; a point mutation of N268Q on Fc was included to make the Agly variant. The genes encoding rCMG2-Fc variants were codon-optimized for expression in *Nicotiana benthamiana*. The full construct consists of the CaMV 35S promoter, Ω leader sequence, gene encoding the Ramy3D signal peptide, followed by rCMG2-Fc gene and octopine synthase terminator (details in Supplementary Figure S1). *Agrobacterium tumefaciens* (*A. tumefaciens*) EHA105 with the helper plasmid (pCH32) was transfected with the resulting binary expression vectors separately *via* electroporation. A binary vector capable of expressing P19 to suppress RNAi-mediated gene silencing in *Nicotiana benthamiana* plants was co-infiltrated with the rCMG2-Fc-APO binary vector as previously described (Arzola et al., 2011).

### Transient Protein Expression in *Nicotiana benthamiana*

Protein was produced through whole-plant agroinfiltration as described previously (Xiong et al., 2018), only differed from the plant age and the *A. tumefaciens* cell densities. Briefly, *A. tumefaciens* strains containing the rCMG2-Fc expression cassette and RNA gene silencing suppressor P19 were suspended into the infiltration buffer (10 mM MES buffer at pH 5.6, 10 mM MgCl_2_ and 150 μM acetosyringone, and 0.02% v/v Silwet-L-77) with a final cell density of 0.25 (A_600_) for each strain. Then, 5-weeks old *Nicotiana benthamiana* plants were vacuum infiltrated with the *A. tumefaciens* suspension for 1 min after vacuum pressure reaches 20 mm inches Hg. Infiltrated plants were incubated at 20°C growth chamber for 6 days allowing protein expression.

### Plant Tissue Collection, Extraction, and Purification

Plant tissue was collected at day 6 after infiltration. To evaluate the average expression level, leaves from 10 plants were collected and stored at −80°C prior to extraction. Leaves were ground to fine powder using mortar and pestle with liquid nitrogen. The leaf powder was weighted and mixed with extraction buffer (1X PBS, 1 mM EDTA, and 2 mM sodium metabisulfite) at a leaf mass (g) to buffer volume (ml) ratio of 1:7. The mixture was incubated on a shaker at 4°C for 1 h and then centrifuged at 1,800*g* at 4°C for 1 h, followed by 0.22 μm filtration to remove insoluble particles. Filtered plant extract was loaded to protein A column and eluted with glycine-HCl buffer at pH of 3.0. Purified protein was immediately titrated to neutral pH with 1 M tris buffer, and buffer exchanged to 1X PBS through overnight dialysis at 4°C.

### ELISA Quantification of rCMG2-Fc in Crude Plant Extracts

Expression of rCMG2-Fc in crude plant extract was quantified by a sandwich ELISA. First, ELISA microplate (Corning, Corning, NY) wells were coated with Protein A (Southern Biotech, Birmingham, AL) at a concentration of 50 μg/ml in 1X PBS buffer for 1 h, followed with plate blocking with 5% nonfat milk in 1X PBS buffer for 20 min. Crude plant extracts and purified standards (Planet biotechnology, Hayward, CA) were loaded to the plate and incubated from 1 h (starting from 0.05 μg/ml, 3-fold serial dilutions). The bound rCMG2-Fc was detected by incubating a horseradish peroxidase (HRP)-conjugated goat anti-human IgG (Southern Biotech, Birmingham, AL) at a concentration of 0.5 μg/ml for 1 h. Plates were washed three times with 1X PBST (1X PBS with 0.05% v/v of Tween20) between each of these steps. All incubation steps were done at 37*°*C, with an incubation volume of 50 μl. Next, 100 μl of ELISA colorimetric TMB substrate (Promega, Fitchburg, WI) was added to each well and incubated for 10 min, followed by the addition of 100 μl of 1 N HCl to stop the reaction. The absorbance at 450 nm was measured with a microplate reader (Molecular Devices, San Jose, CA). The absorbance of protein standard was plotted as a function of rCMG2-Fc concentration, and was fitted to the 4-parameter model in SoftMax Pro software. The concentration of rCMG2-Fc in crude plant extract was determined by interpolating from the linear region of the standard curve.

### SDS-PAGE and Western Blotting

SDS-PAGE and Western blot analyses were performed on purified (protein A) rCMG2-Fc variants. Protein was denatured and reduced by treating samples at 95*°*C for 5 min with 5% (v/v) of 2-mercaptoethanol (Sigma-Aldrich, St. Louis, MO). For nonreducing SDS-PAGE, samples were denatured by heat treatment at 95*°*C for 5 min. Samples were loaded to precast 4–20% SDS-Tris HCl polyacrylamide gels (Bio-Rad Laboratories, Hercules, CA), running at 200 V for 35 min. For SDS-PAGE, the gel was washed three times with water and stained with Coomassie Brilliant Blue R-250 Staining Solution (Bio-Rad Laboratories, Hercules, CA). For Western blot analysis, samples were transferred to a nitrocellulose membrane by electrophoretic transfer using the iBlot Gel Transfer Device (ThermoFisher, Waltham, MA). For Western blot detecting the CMG2 domain, the membrane was probed with a goat anti-CMG2 polyclonal antibody (ThermoFisher, Waltham, MA) at a concentration of 0.3 μg/ml, followed by incubation of a polyclonal AP-conjugated rabbit anti-goat IgG antibody (Sigma-Aldrich, St. Louis, MO) at 1:10,000 dilution. For Western blot detecting the Fc domain, the membrane was incubated with a polyclonal AP-conjugated goat anti-human IgG antibody (Southern Biotech, Birmingham, AL) at 1:3,000 dilution. The blots were developed using SIGMAFAST BCIP/NBT (Sigma-Aldrich, St. Louis, MO) according to the product instruction.

### SDS-PAGE Densitometry

CMG2-Fc standard from Planet Biotechnology (0.5, 0.75, 1.0, 1.25, and 1.5 μg/lane) and rCMG2-Fc variants (APO, ER, and Agly) were reduced, denatured, and run on a 4–12% Bis-Tris gel (Invitrogen, Carlsbad, CA) gel at 50 mA for 1.5 h. After staining for 1 h with Coomassie Brilliant Blue R-250 staining solution (Bio-Rad Laboratories, Hercules, CA), the gel was washed with water overnight. Next morning, the gel was scanned with Gel Doc™ XR+ System (Bio-Rad laboratories, Hercules, CA), and a standard curve was established by plotting total protein mass of standards as a function of band intensity. Then, the band intensity for the ~50 kDa band of rCMG2-Fc variants was interpolated onto the standard curve to determine the mass of intact rCMG2-Fc, and calculate their concentrations.

### Protein Sequence Identification by Liquid Chromatography-Tandem Mass Spectrometry (LC-MS/MS)

Purified rCMG2-Fc variants were subjected to protein sequence identification by mass spectrometry (LC-MS/MS). First, 10 μg of purified rCMG2-Fc variants were subjected to SDS-PAGE analyses under reducing conditions as described in SDS-PAGE and Western Blotting section. After staining the gel in Coomassie Brilliant Blue R-250 (Bio-Rad, Hercules, CA, USA) and rinsing in water, the rCMG2-Fc protein band was excised from the gel and submitted to the Proteomics Core facility of University of California, Davis for LC-MS/MS-based protein identification. Briefly, the protein was digested with sequencing grade trypsin per manufacturer’s recommendations (Promega, Madison, WI, USA). Specific conditions can be found on UC Davis Proteomics Core Facility website^1^ (“Ingel Digestion Protocol 2”). Peptides were dried using vacuum concentrator and resolubilized in 2% acetonitrile/ 0.1% trifluoroacetic acid. Peptides were analyzed by LC-MS/MS on a Thermo Scientific Q Exactive Orbitrap Mass Spectrometer in conjunction Proxeon Easy-nLC II HPLC and Proxeon nanospray source. The digested peptides were loaded on a Magic C18 200 Å 3 U reverse phase column (75-micron × 150 mm) and eluted using a 90-min gradient with a flow rate of 300 nl/min. An MS survey scan was obtained for the m/z range 300–1,600, spectra of MS/MS were developed using a top 15 method. An isolation mass window (2.0 m/z) was used for the precursor ion selection, and normalized collision energy (27%) was used for fragmentation. Tandem MS spectra were extracted and charge state deconvoluted by Proteome Discoverer (Thermo Scientific, Asheville, NC, USA). The MS/MS samples were analyzed using X! Tandem (The GPM, thegpm.org; version TORNADO (2013.02.01.1)). X! Tandem was set up to search UniProt-Nicotiana benthamiana_database (20140416, 1,538 entries), the cRAP database of common laboratory contaminants^2^ (114 entries) plus an equal number of reverse protein sequences assuming the trypsin enzyme digestion. Scaffold Proteome Software version 4.0.6.1 (OR, USA) was used to confirm protein identifications. X! Tandem identifications required at least –Log (Expect Scores) scores of greater than 1.2 with a mass accuracy of 5 ppm. Protein identifications were accepted if they contained at least two identified peptides. Using the parameters above, the Decoy False Discovery Rate (FDR) was calculated to be 4.5% on the protein level and 1.94% on the spectrum level. Proteins that contained similar peptides and could not be differentiated based on MS/MS analysis alone were grouped to satisfy the principles of parsimony.

### Protein N-Glycoform Analysis by Dynamic Multiple Reaction Monitoring

rCMG2-Fc protein dissolved in 50 mM NH_4_HCO_3_ was denatured with 2 μl of dithiothreitol (DTT) in a 65°C water bath for 50 min, followed by the alkylation with 4 μl of iodoacetamide (IAA) in the dark for 20 min. The protein was then digested with 1 μg of trypsin in a 37°C water bath for 18 h. After the digestion, the mixture was frozen at −20°C for 1 h to deactivate the trypsin. For N-glycosylation analysis, 2 μl of the mixture was separated with an Agilent Eclipse plus C18 column (RRHD 1.8 μm, 2.1 mm × 150 mm) coupled to an Agilent Eclipse plus C18 guard column (RRHD 1.8 μm, 2.1 mm × 5 mm), using a 10-min-gradient where solvent A with 0.1% formic acid (FA) and 3% of ACN in water, and solvent B with 0.1% of FA and 90% of ACN in water were used for separation. The analysis was conducted on an Agilent 1290 infinity ultra-high-pressure liquid chromatography (UHPLC) system coupled to an Agilent 6495 triple quadrupole (QQQ) mass spectrometer, which was operated in a dynamic multiple reaction monitoring (dMRM) mode. The glycosylation site of the protein is on the infused IgG Fc region so that the transition list used here was adapted from the dMRM method of serum IgG, the development of which was described in great details in the study conducted by Hong et al. (2013). To modify the method for rCMG2-Fc glycosylation quantitation, the plant N-glycan compositions containing xylose rather than sialic acid were used. In total, nearly 30 unique transitions for targeted glycopeptides and peptides of the rCMG2-Fc protein composed of the precursor ion, the product ion, the collision energy, and the retention time for each individual compound of the protein were developed for the dMRM method. The targeted glycopeptides were selected as precursor ions and several common oxonium fragments with m/z values as 204.08 and 366.14 were used as product ions. The software used for data analysis was Agilent MassHunter Quantitative Analysis B.05.02 software. To calculate the relative abundance of each glycopeptide, the abundance of individual glycopeptide was normalized to the abundance of the quantitating peptide.

To validate the glycopeptides quantitated with the dMRM method, glycoproteomic analysis was conducted on rCMG2-Fc proteins. After the trypsin digestion of protein samples, glycopeptides were enriched with iSPE-HILIC cartridges. Then enriched samples were dried completely and reconstitute with 30 μl of water for LC-MS/MS analysis. One microgram of sample was separated with a Thermo Acclaim PepMap RSLC C18 column using a 180-min gradient. The analysis was conducted on a Thermo UltiMate 300 nano LC system coupled to an Orbitrap Fusion Lumos Tribrid mass spectrometer. The collected raw data were inspected with the software FreeStyle and the MS/MS spectra were search with the software Byonic.

### Toxin Neutralization Assay

Cell viability was determined by the MTS [3-(4,5-Dimethylthiazol-2-yl)-5-(3-carboxymethoxyphenyl)-2-(4-sulfophenyl)-2H-tetrazolium, inner salt] assay. Mouse macrophage cells RAW264.7 (ATCC, Manassas, VA) were seeded at 2*10^4^/well on 96-well cell culture plates in Dulbecco’s modified Eagle medium (Corning, Corning, NY) supplemented with 5% heat-inactivated fetal bovine serum (VWR, Radnor, PA) and 2mM of GlutaMAX (Thermo Fisher, Waltham, MA) for 17 hours in a cell incubator (7% CO_2_, 37°C). Toxin neutralization efficacy was measured by assessing cell viability with 1.4X serial dilutions of rCMG2-Fc variants in the presence of a constant amount of LT (PA at 100 ng/ml, LF at 200 ng/ml). rCMG2-Fc serial dilutions were incubated with LT for 30 min at 37*°*C, and then the mixtures were transferred to the cell plates, followed by a 4-h incubation at 37*°*C, 7% CO_2_. MTS reagent (Promega, Fitchburg, WI) was added to the wells at 20 μl/well, following with another 4-h incubation at 37*°*C, 7% CO_2_. For TNAs with Fc gamma receptors blocked, cells were first treated with 2.4G2 antibody (BD Biosciences, San Jose, CA) at the concentration of 5 or 10 μg/ml for 15 min, then proceeded to the TNA as described above. The plates were gently mixed to ensure uniform color distribution in the wells, and then the absorbance at 490 nm was read with a 96-well plate reader (Molecular Devices, San Jose, CA). The EC_50_s were calculated using GraphPad Prism.

### Biolayer Interferometry Analysis

The binding between rCMG2-Fc variants and PA was measured in real time by biolayer interferometry (BLI) using an Octet RED384 instrument (ForteBio, Fremont, CA). rCMG2-Fc in 1X PBS buffer with 1 mM MgCl_2_ was captured on Anti-hIgG Fc Capture Biosensors (ForteBio, Fremont, CA) at the surface density resulting in a wavelength shift between 0.8 and 1 nm. PA at known concentrations (starting from 5.4 μg/ml, 2X serial dilutions) were loaded to the sample plate. Sensors loaded with rCMG2-Fc were dipped into PA solutions in the kinetics buffer (ForteBio, Fremont, CA) for 300 s, and then switched to the kinetics buffer (ForteBio, Fremont, CA) for 600–900 s allowing for dissociation. The sensorgrams were fitted with 1:1 binding model using ForteBio Data Analysis software (ForteBio, Fremont, CA).

### Functional rCMG2-Fc (PA Binding) ELISA

The microplate (Corning, Corning, NY) wells were coated with 100 μl of PA of anthrax (BEI resources, Manassas, VA) at concentration of 2.5 μg/ml for 1 h in 1X PBS buffer, and then the plate was blocked with 5% nonfat milk for 30 min. The rCMG2-Fc samples in 1X PBS with 1 mM MgCl_2_ (controls and 37°C incubated samples) were loaded to the plate, 100 μl per well (starting from 2.5 μg/ml, 2.5X serial dilutions). The functional rCMG2-Fc bound to the PA was detected with an HRP-conjugated goat anti-human IgG (Southern Biotech, Birmingham, AL) at concentration of 0.5 μg/ml for 1 h. Plates were washed for three times with 1X PBST between steps above, and all the steps were done at room temperature. The 37°C incubation was eliminated to avoid potential effects on protein activity. The plate was developed with 100 μl of TMB substrate (Promega, Fitchburg, WI) and stopped by the 100 μl of 1 N HCl. The absorbance at 450 nm was read with a microplate reader (Molecular Devices, San Jose, CA).

### Molecular Dynamics Simulation of rCMG2-Fc Glycoforms: Construction of Initial Protein Configurations

The atomic coordinates of the von Willebrand factor A domain of anthrax toxin receptor capillary morphogenesis protein 2 (CMG2) were obtained from the protein data bank with 4.3 Å resolution X-ray crystallography (PDB ID: 1TZN) (Lacy et al., 2004). The atomic coordinates of the fragment crystallizable (Fc) region of human Immunoglobulin G were obtained from the protein data bank with 2.2 Å (PDB ID: 3SGJ) (Ferrara et al., 2011). Missing residues, de-mutations, and fusion of the two crystal structures with the IgG2 hinge linker was performed with Modeller 9.16 (Webb and Sali, 2014). All four linker cysteines were modeled to participate in symmetric inter-chain disulfide bonds. Aglycosylated (Agly), GnGnXF, and MAN8 glycoforms of the chimeric dimer CMG2-Fc were simulated for 100 ns. In molecular simulations, we used modified nomenclatures to specify the simulated glycoform as only the predominant glycoform from each variant was simulated.

### Glycan Attachment

Glycans for the GnGnXF, and MAN8 glycoforms were attached to Asn_268_ for both monomers. Glycans were attached using the glycam.org glycoprotein builder (Woods, 2005). The glycans were subsequently sequentially energy minimized through rigid rotation about three bonds: N_γ_^prot^-C_1_^glycan^, C_β_^prot^-C_γ_^prot^, and C_α_^prot^-C_β_^prot^ using the minimization scheme outlined in Bernardi et al. (2017). Energy was calculated with GROMACS 5.1.4 (Bekker et al., 1993; Berendsen et al., 1995; Abraham et al., 2015) using the AMBER ff14SB (Maier et al., 2015) and GLYCAM06-j (Kirschner et al., 2008) force fields. The lowest energy rotational conformer was selected for the initial coordinates of the molecular dynamics simulation for each glycoform.

### Simulation Setup

About 100 ns simulations of Agly, MAN8, and GnGnXF rCMG2-Fc glycoforms were performed in GROMACS with the AMBER ff14SB and GLYCAM06-j force fields. The AMBER topology files were exported to GROMACS format using ACPYPE (da Silva and Vranken, 2012) with updated modifications which enable simulations with the GLYCAM forcefield in GROMACS (Bernardi et al., 2019). The 100 ns production simulations were first preceded by energy minimization in vacuum, solvation, solvated energy minimization, a 100 ps NVT equilibration, and finally a 100 ps NPT equilibration. Both energy minimizations were terminated with a maximum force tolerance of 1,000 kJ mol^−1^ nm^−1^. Each glycoform was solvated with explicit water with a minimum distance of 1.2 nm between the glycoprotein and the edge of the periodic box. The solvated systems were then neutralized with either sodium or chloride ions, and then concentrated to 0.155 M NaCl. The velocity-rescale thermostat (Bussi et al., 2007) was used with a reference temperature of 310 K and a time constant of 0.1 ps. The isotropic Parrinello-Rahman (Parrinello and Rahman, 1981) barostat was used with a reference pressure of 1 bar, a time constant of 2 ps, and an isothermal compressibility of 4.5 × 10^−5^ bar^−1^. All nonbonded interactions employed a short-range cutoff of 1 nm, with vertically shifted potentials such that the potential at the cutoff range is zero. The Particle-Mesh Ewald method (Darden et al., 1993) with cubic interpolation was used to model long range electrostatic interactions. All non-water bonds were constrained with LINCS (Hess, 2008), while water bonds were constrained with SETTLE (Miyamoto and Kollman, 1992). A 2 fs timestep was used with a sampling interval of 0.1 ns, for a total of 1,000 data points per 100 ns simulation.

## Result

### Transient Expression of rCMG2-Fc Variants

**Figure 1:**
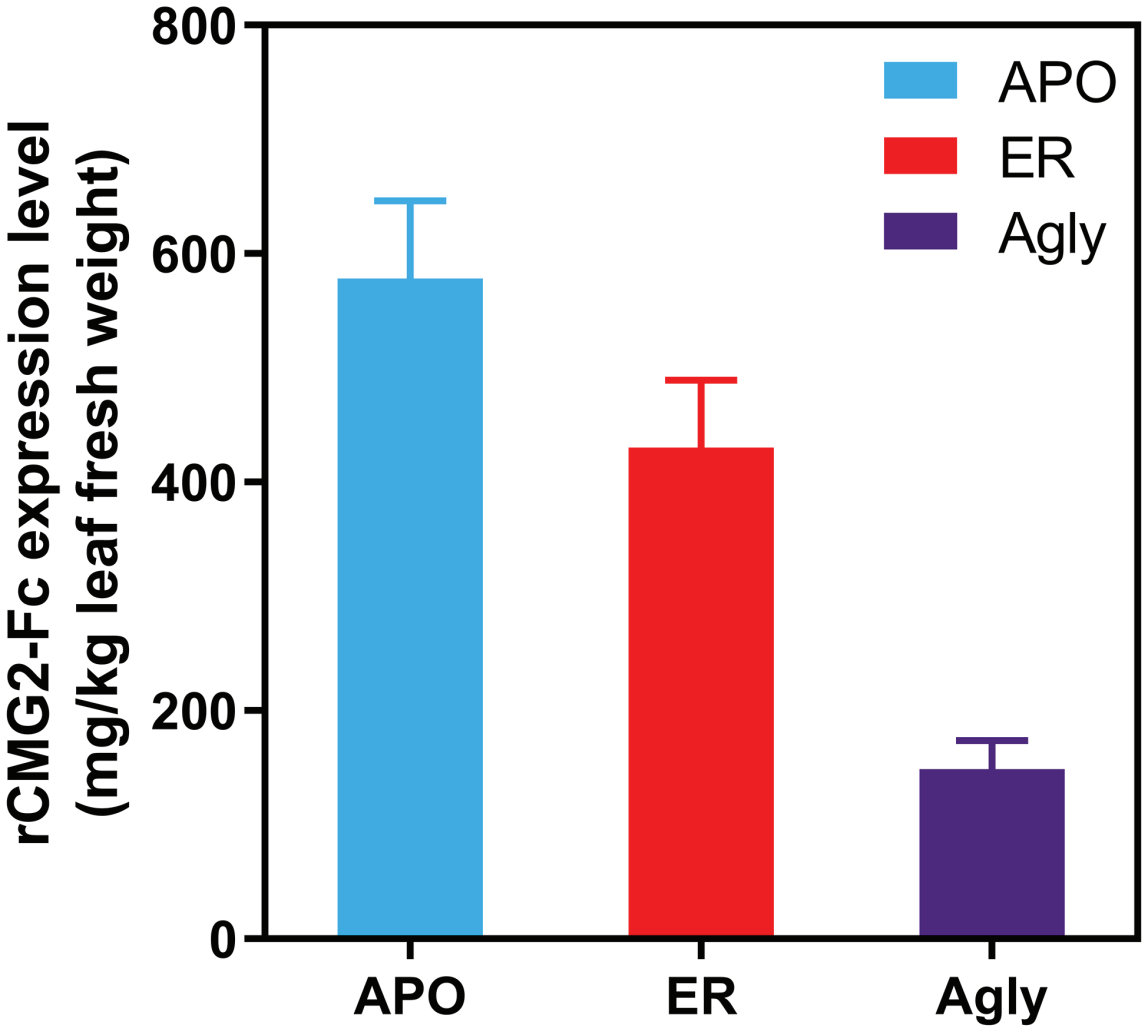
Recombinant CMG2-Fc levels in crude plant extracts at 6 dpi. The expression level rankings from high to low are: APO, ER, and Agly variants. Error bars represent standard error of two infiltration batches (Leaves from 10 plants per batch).

Recombinant CMG2-Fc variants were transiently expressed in *Nicotiana benthamiana* whole plants *via* agroinfiltration under identical conditions, and the expression levels were determined in crude leaf extract at 6 days post infiltration (dpi) with a sandwich ELISA detecting the Fc domain of rCMG2-Fc. The expression level rankings on the leaf fresh weight basis (LFW) from high to low were: APO (578 mg/kg LFW), ER (430 mg/kg LFW), and Agly (148 mg/kg LFW) variants (Figure 1). Both APO and Agly variants, which only differ in the N-glycosylation generated by a point mutation of N268Q, were targeted to plant Apoplast. The only N-glycosylation site within rCMG2-Fc is located at the CH2 domain of IgG1 Fc (N_297_ in IgG1, N_268_ in rCMG2-Fc). The significantly higher expression of the APO variant with respect to the Agly variant might be due to stabilizing effects of N-glycans on protein accumulation *in planta*. This observation is consistent with previous studies, where proteins are more susceptible to protease cleavage after deglycosylation (Liu et al., 2008; Zheng et al., 2011). In many cases, targeting proteins to the ER will result in a greater protein yield compared with targeting to the cytosol or apoplast (Pan et al., 2008; Sainsbury and Lomonossoff, 2008; Pillay et al., 2014). The plant apoplast is usually not a preferred location for recombinant protein accumulation given the abundance and poor specificity of proteases (Benchabane et al., 2008; Pillay et al., 2014). However, in this case, the APO variant resulted in a high accumulation, which indicates that rCMG2-Fc is stable even in a protease-rich environment when glycosylated. Comparing the APO to ER variants, the expression levels are similar. Besides the contribution from the high stability of rCMG2-Fc in apoplast, it is also possible that the ER variant was under-extracted due to the additional ER membrane barrier considering that no detergent was used in the extraction buffer.

**Figure 2:**
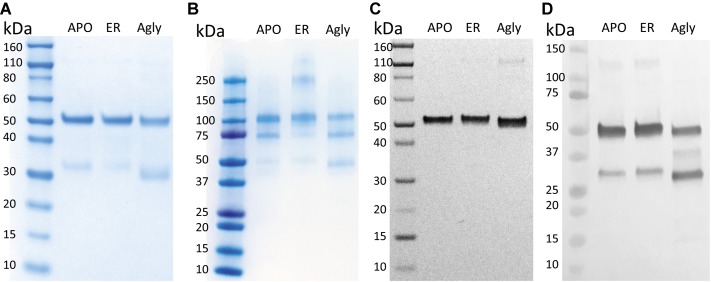
SDS-PAGE and Western Blots of rCMG2-Fc variants. For SDS-PAGE, **(A)** ~1 μg of total soluble protein/lane, run under reducing and denaturing conditions; **(B)** ~1 μg of total soluble protein/lane, run under nonreducing and denaturing conditions. For Western blots, **(C)** ~100 ng of total soluble protein/lane, run under reducing and denaturing conditions, detecting CMG2 domain; **(D)** ~500 ng of total soluble protein/lane, run under reducing and denaturing conditions, detecting Fc domain.

In Figure 2A, purified rCMG2 of all variants showed a dominant band around ~50 kDa, corresponding to the rCMG2-Fc monomer, the faint bands below were likely a Fc-containing fragment. This observation is consistent with the hypothesis that N-glycosylation stabilizes protein *in planta*, as less degradation was found in APO and ER variants than in the Agly variant. It was hypothesized that all three variants were degraded at the same site(s) as proteolytic cleavage shows some degree of site specificity. By N-terminal sequencing of the Fc-containing fragment, three cleavage sites were identified within or near the linker of rCMG2-Fc, and those sites were shared among variants (Supplementary Figure S2). It is not surprising that cleavage occurred near the linker region because the linker in rCMG2-Fc is flexible and proteases tend to cleave at solvent-exposed, flexible and less structured regions (Song et al., 2012). The SDS-PAGE under nonreducing conditions (Figure 2B) reveals that the expressed rCMG2-Fc primarily formed a homodimeric species (~100 kDa). The lower bands were dimerized Fc-containing fragments. The band at 250 kDa in the ER variant sample might represent protein aggregate, which was not observed in APO or Agly variants, suggesting that the ER variant is prone to aggregation more than the other two variants. Western blots detecting CMG2 (Figure 2C) and Fc (Figure 2D) were conducted to confirm the presence of both domains. These two blots confirm the existence of both domains, and also confirm that the lower band in Figure 2A only contains an Fc fragment. No detectable CMG2 fragment in Figure 2C confirms that the protein degradation happened during *in planta* production, and rCMG2-Fc remained intact during storage once being purified from crude plant extract.

### rCMG2-Fc Amino Acid Sequence Determination

**Figure 3:**
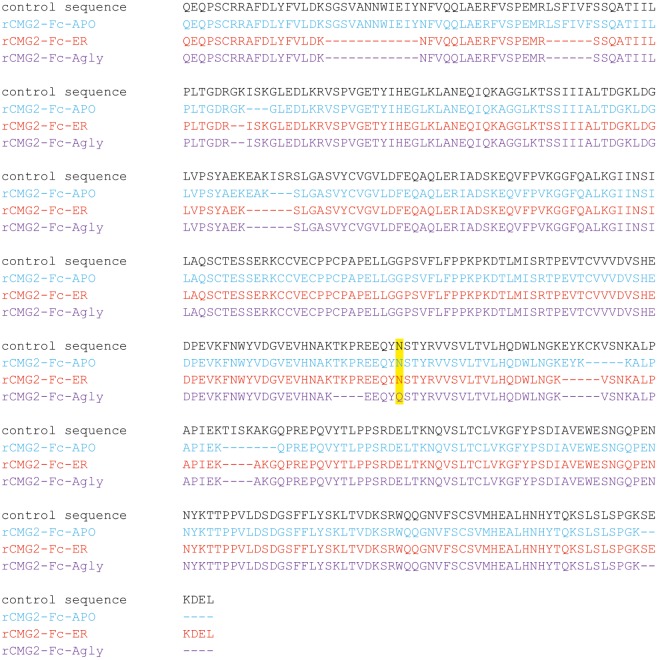
Amino acid sequences of the rCMG2-Fc variants compared to the control (theoretical) sequence. Variant coloring: APO (blue), ER (red), and Agly (purple).

To confirm the amino acid sequence of rCMG2-Fc variants, purified variants were subjected to LC–MS/MS analysis. The N-glycosylation site is highlighted in yellow, and the sequences not clearly detected are represented with dashes (Figure 3). The point mutation (N268Q) in Agly variant was detected as predicted. The sequence coverage with respect to the control (theoretical) sequence for the APO, ER, and Agly variants were 95.7, 91.7, and 90.6%, respectively. The high sequence coverage with both N- and C-terminal predicted sequences confirmed the production of full length rCMG2-Fc variants in the plants (Figure 3; Supplementary Figure S3).

### Mass Spectrometry Analysis of rCMG2-Fc N-Glycosylation Profile

**Figure 4:**
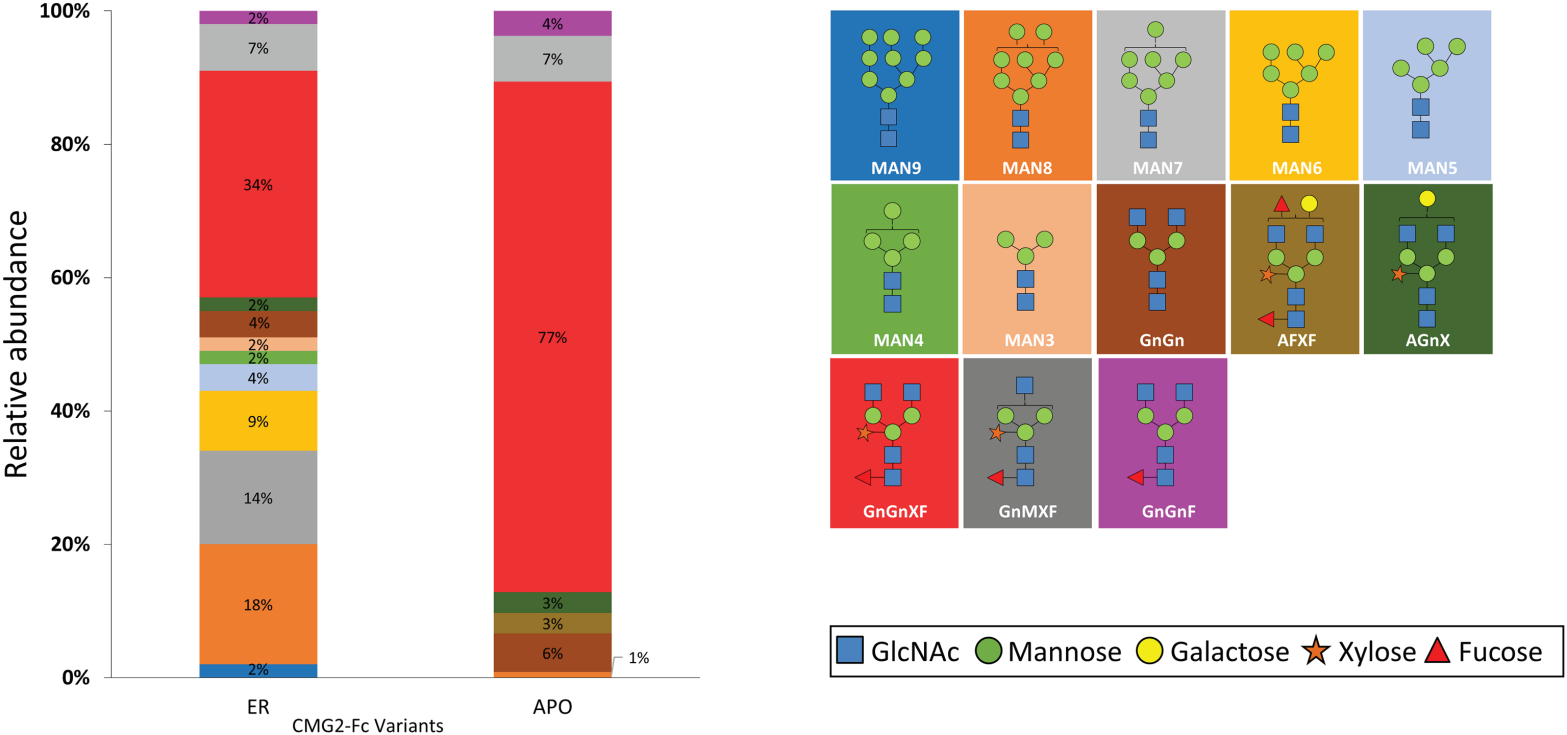
N-glycosylation composition of rCMG2-Fc variants (ER and APO). The relative abundance of each glycoform is represented as the percentage of total signal. The schematic representation and the name of each glycan are listed to the right of the N-glycosylation profile figure, with background color matching with the corresponding glycan.

To determine the glycoform profile, rCMG2-Fc variants were subjected to LC-MS/MS analysis for N-glycosylation identification. For the APO variant, 99% of N-glycoforms were plant complex-type, with the most abundant structure of GnGnXF (Figure 4; Supplementary Figure S4), indicating that protein went through the secretory pathway and was fully glycosylated as expected. For the ER variant, the relative abundance of mannose-type N-glycans was 50%, with MAN8 (18%) as the most abundant mannose-type structure (Figure 4; Supplementary Figure S4). Overall, a complex-type N-glycan (GnGnXF) was the most predominant N-glycan (34%). To validation the dMRM methods, both APO and ER variants were subjected to glycoproteomic analysis. Compounds quantitated in dMRM methods were identified in glycoproteomic analysis, and several representative full MS spectra for the APO and ER variants are shown in Supplementary Figures S5A,B, respectively, with the compounds assigned to peaks. Comparing the APO to ER variant, a significant shift from plant complex-type to mannose-type N-glycans was observed, where about half of rCMG2-Fc was retained in ER upon the addition of C-terminal ER retention sequence SEKDEL. Although the retention is not perfect, the glycoform profiles of APO and ER variants are distinct. This incomplete ER retention agrees with previous studies (He et al., 2012; Roychowdhury et al., 2018), where proteins can sometimes escape the ER retention signal and progress to downstream N-glycosylation processes. The glycan MS data was used to select representative glycoforms for molecular dynamics simulations.

### Toxin Neutralization Assay

**Figure 5:**
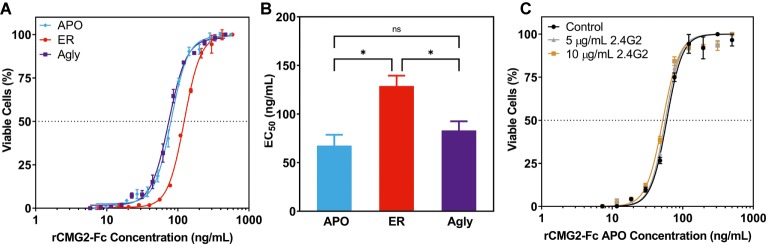
Neutralization of anthrax lethal toxin by rCMG2-Fc variants. **(A)**: rCMG2-Fc variants at various concentrations were incubated with 100 ng/ml of PA and 200 ng/ml of LF for 30 min at 37°C prior to adding to the mouse macrophage cell culture (RAW264.7). Cell viability was measured with an MTS assay. Data shown are from one representative experiment out of three replications. Each data point corresponds to the mean of duplicate wells on the sample plate, and error bars represent standard error of the mean. **(B)**: TNA EC_50_ values for rCMG2-Fc variants (*: *p* < 0.05, ns: *p* > 0.05; Tukey’s test performed after observing a significant difference (*p* = 0.0009) in one-way ANOVA.) Each bar represents the mean from three separate experiments with duplicate well measurements, with error bars representing standard deviation from the means. **(C)**: Toxin neutralization assay using the APO variant with FcγR blocked. Control: without anti-FcγR antibody treatment. Two concentrations of anti-FcγR antibody were tested: 5 and 10 μg/ml, which were incubated with macrophages for 15 min prior to rCMG2-Fc addition. EC_50_ values for all three conditions were comparable.

To test the toxin neutralization efficacy of rCMG2-Fc in a biologically relevant environment, a cell-based toxin neutralization assay (TNA) was developed using a mouse macrophage cell line (RAW264.7). Since toxin concentrations reported in the literature vary, the concentrations and ratio of PA and LF were optimized by toxin titration. A PA concentration of 100 ng/ml and an LF concentration of 200 ng/ml were chosen, as these concentrations resulted in almost complete cell killing (97%, Supplementary Figure S6). Once the lethal toxin (LT, the combination of PA and LF) concentration was fixed, the toxin neutralization efficacy of rCMG2-Fc variants was analyzed over a range of rCMG2-Fc concentrations. The concentration of intact rCMG2-Fc was determined by SDS-PAGE densitometry (Supplementary Figure S7). The dose-response curves are shown in Figure 5A, from which the EC_50_ values were determined. The average EC_50_ values from low to high are, 67.6 ng/ml for the APO variant, 83.15 ng/ml for the Agly variant, and 128.9 ng/ml for the ER variant, where the EC_50_ of the ER variant was statistically different (Figure 5B) from the Agly and APO variants (*p* < 0.05).

This difference in EC_50_ could have resulted from the toxin neutralization that depended on the Fc gamma receptors (FcγR) on macrophages, where the interaction between Fc and FcγR on the cell surface contributes to toxin neutralization, which in turn lowers the EC_50_ (Verma et al., 2009; Ngundi et al., 2010). Thus, TNAs with FcγR blocking were performed to examine the possible contribution of the FcγR. Cells were pre-treated with anti-FcγR antibody 2.4G2 for 15 min prior to the TNA. The resulting dose-response curves are shown in Figure 5C; Supplementary Figure S8, where the blocked FcγR curves overlap with the control curve (no antibody), demonstrating that the FcγR-Fc interaction did not contribute to toxin neutralization. The differences in EC_50_ values could result from differences in binding kinetics between rCMG2-Fc variants and PA, stability of rCMG2-Fc variants during cell culture incubation, or rCMG2-Fc variant conformation. Additional experiments including BLI, functional ELISA and MD simulation were conducted to evaluate these possibilities.

### Binding Kinetics Between rCMG2-Fc and PA

**Figure 6:**
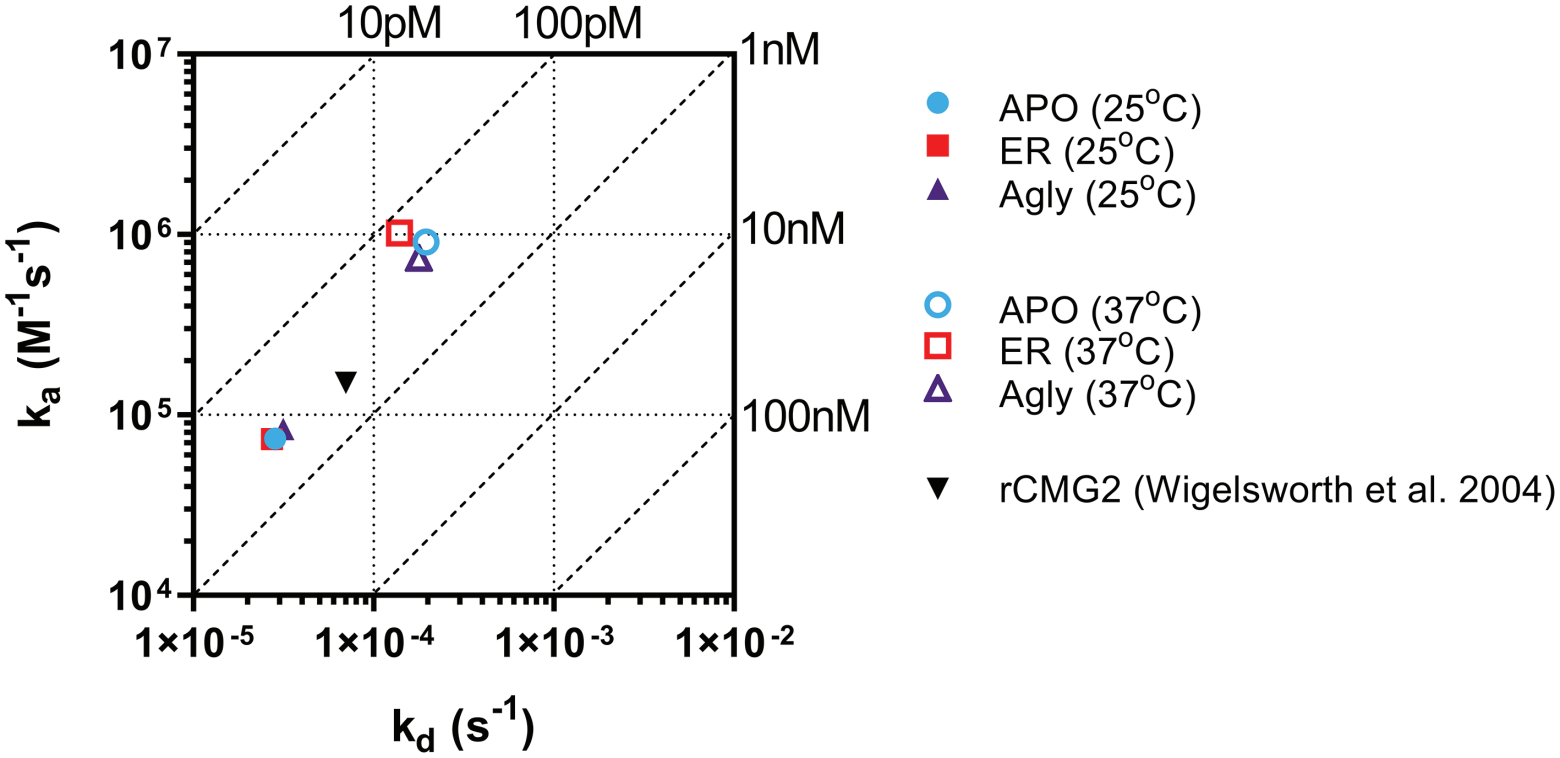
Binding and dissociation rate constants of rCMG2-Fc variants to PA measured at 25 and 37°C were calculated by fitting sensorgrams (Supplementary Figure S9) to a Langmuir 1:1 interaction model. The *k_a_* and *k_d_* values are presented in an on-off rate map with diagonal lines indicating the equilibrium dissociation constant (*K_D_*). Published binding and dissociation kinetic parameters (Wigelsworth et al., 2004) between rCMG2 and PA are included for comparison.

The binding kinetics between rCMG2-Fc variants and PA were measured in real-time with BLI. The measurements were taken at room temperature (25*°*C) and body temperature (37*°*C) to provide information on how binding kinetics are affected by temperature, and potentially explains the difference in EC_50_ values. Comparing binding kinetics between rCMG2-Fc variants and PA at the same temperature, we found the association rate constant (*k_a_*) and the dissociation rate constant (*k_d_*) to be similar regardless of protein N-glycosylation (Figure 6). BLI sensorgrams and fitting curves are shown in Supplementary Figure S9. As temperature was increased from 25 to 37*°*C, higher *k_a_* and *k_d_* values were obtained (Figure 6) as expected, since both binding and dissociation will happen faster at an elevated temperature. The equilibrium dissociation constant (*K_D_*) at both temperatures is on the same order (100 pM–1 nM), with slightly lower *K_D_* at 37*°*C, demonstrating a desired strong binding between rCMG2-Fc and PA (Table 1). These results show that all rCMG2-Fc variants are functional with very high binding affinities to PA, and the binding equilibrium is not strongly affected by temperature. Comparing with previously reported binding kinetics of rCMG2 to PA (Wigelsworth et al., 2004), both *k_a_* and *k_d_* (Figure 6) are in agreement with our BLI results. Moreover, protein N-glycosylation showed no significant impact on binding kinetics of rCMG2-Fc to PA, as the average deviations in *K_D_* values were observed to be 2% (25*°*C) and 26% (37*°*C), which is considered to be within the experimental error (30%) of this method (Kamat and Rafique, 2017).

**Table 1:** Summary of *K_D_* values at 25 and 37°C.

Variants	*K_D_* (M) at 25°C	*K_D_* (M) at 37°C
APO	3.9 × 10^−10^	2.1 × 10^−10^
ER	3.8 × 10^−10^	1.5 × 10^−10^
Agly	3.8 × 10^−10^	2.4 × 10^−10^
rCMG2*	4.0 × 10^−10^	NA

* *Published *K_D_* (Wigelsworth et al., 2004) between rCMG2 and PA is included in the last row for comparison*.

### Thermostability of rCMG2-Fc

Protein stability is an important property of biopharmaceuticals, as it is often required that a protein remains stable and active during both storage and circulation in the target system after injection. To assess the stability of rCMG2-Fc variants and understand the EC_50_ differences observed in TNAs, a functional ELISA was developed to measure the amount of active rCMG2-Fc (able to bind to PA) after incubation at 37*°*C for a range of time periods. Four time periods were tested: 1, 2, 3 h and overnight (20 h). Since all three variants showed similar binding kinetics in BLI experiments, we hypothesized that the differences in EC_50_ may result from rCMG2-Fc stability differences between variants during the 8-h cell incubation period at 37*°*C. With a less stable variant, the fraction of functional rCMG2-Fc decays over time, together with the fact that CMG2 and PA binding is reversible (Wigelsworth et al., 2004), resulting in a higher EC_50_ compared to more stable variants.

**Figure 7:**
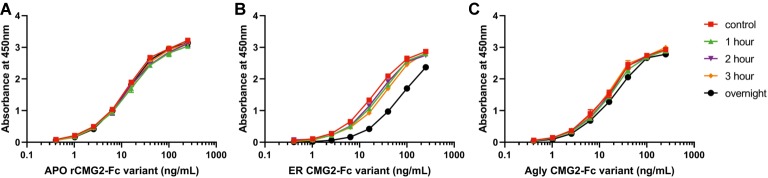
Functional ELISA results for the APO **(A)**, ER **(B)**, and Agly **(C)** rCMG2-Fc variants. Variants were diluted to 2.5 μg/ml and incubated at 37°C for 1, 2, 3 h and overnight prior to ELISA quantification. Controls were kept at 4°C prior to ELISA. Variants were serially diluted and added to the enzyme immunoassay plate precoated with PA. Binding was measured by an ELISA detecting the Fc region. Error bars are smaller than the markers, thus are omitted. All sample treatments were done simultaneously under identical conditions.

The observed binding to PA was the same before and after incubation at 37*°*C for APO and Agly variants (Figures 7A,C). For the ER variant, the fraction of functional rCMG2-Fc decayed over time, and a significant drop was observed for the overnight incubation sample (Figure 7B), which explains the higher EC_50_ for the ER variant in the TNA (Figure 5B). The thermostability result is in agreement with the TNA results, with stability rankings from high to low: APO/Agly then ER variants.

### Molecular Dynamics Simulation of rCMG2-Fc Glycoforms

Molecular dynamics (MD) simulations of the respective predominant glycoform in the three rCMG2-Fc variants were performed to obtain high resolution structural and dynamical information. The three simulated glycoforms were GnGnXF, MAN8, and Agly. These glycoforms were selected according to the mass spectrometry analysis, shown in Figure 4. GnGnXF is the predominant glycoform of both the APO and ER variants. Therefore, the MAN8 glycoform, the second-most expressed glycan in the ER variant, was simulated to elucidate differences between the APO and ER variants.

### Macrostructural Analysis

**Figure 8:**
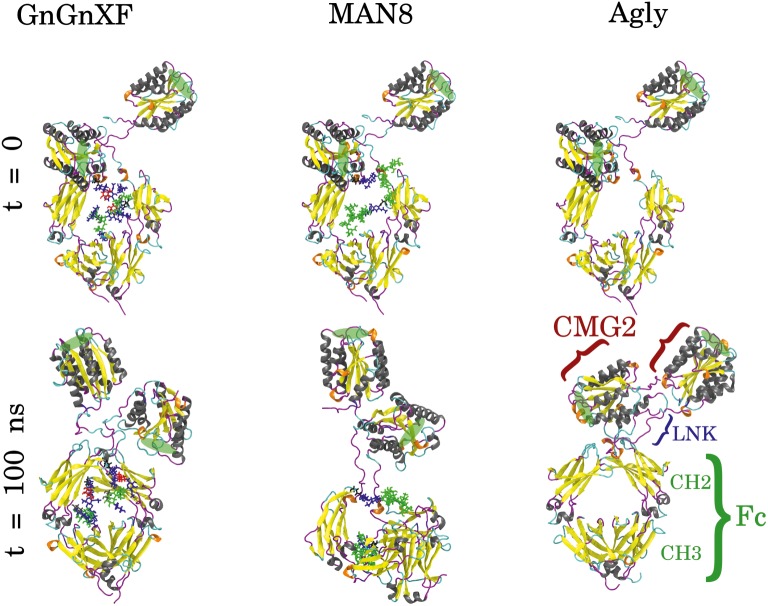
Initial (*t* = 0) and final (*t* = 100 ns) conformations of GnGnXF, MAN8, and Agly rCMG2-Fc. The N-terminal CMG2 portion is oriented at the top of each image, and the C-terminal Fc portion is oriented at the bottom, covalently joined to the CMG2 portion by a flexible linker (LNK). The glycans are shown in licorice representation, attached to the Fc regions at Asn_268_. The protein secondary structure is colored as follows: α-helix (gray), β-sheet (yellow), 3_10_-helix (orange), turn (cyan), and coil (magenta). The PA binding sites are highlighted with green ellipses.

GnGnXF, MAN8, and Agly rCMG2-Fc glycoforms were each independently simulated for 100 ns. Figure 8 shows the initial and final conformations of all glycoforms from the simulations. The images at *t* = 0 are all aligned by the Fc CH2 domain; the images at *t* = 100 ns are each independently aligned to best illustrate macrostructural orientation. For all simulations, we see the core structure of the protein remains intact. However, there is significant variability in the macrostructural orientation of the final structures’ CMG2 and Fc domains. The final GnGnXF and Agly structures exhibit significantly contracted linkers with respect to the final MAN8 structure, where the linker is fully extended. All glycoforms retain accessibility of the PA binding site after simulation. This is in agreement with the BLI (Figure 6), where all three variants have similar *k_a_* and *k_d_*, and the functional ELISA (Figure 7), where all three variants have control curves with similar absorbance level.

**Figure 9:**
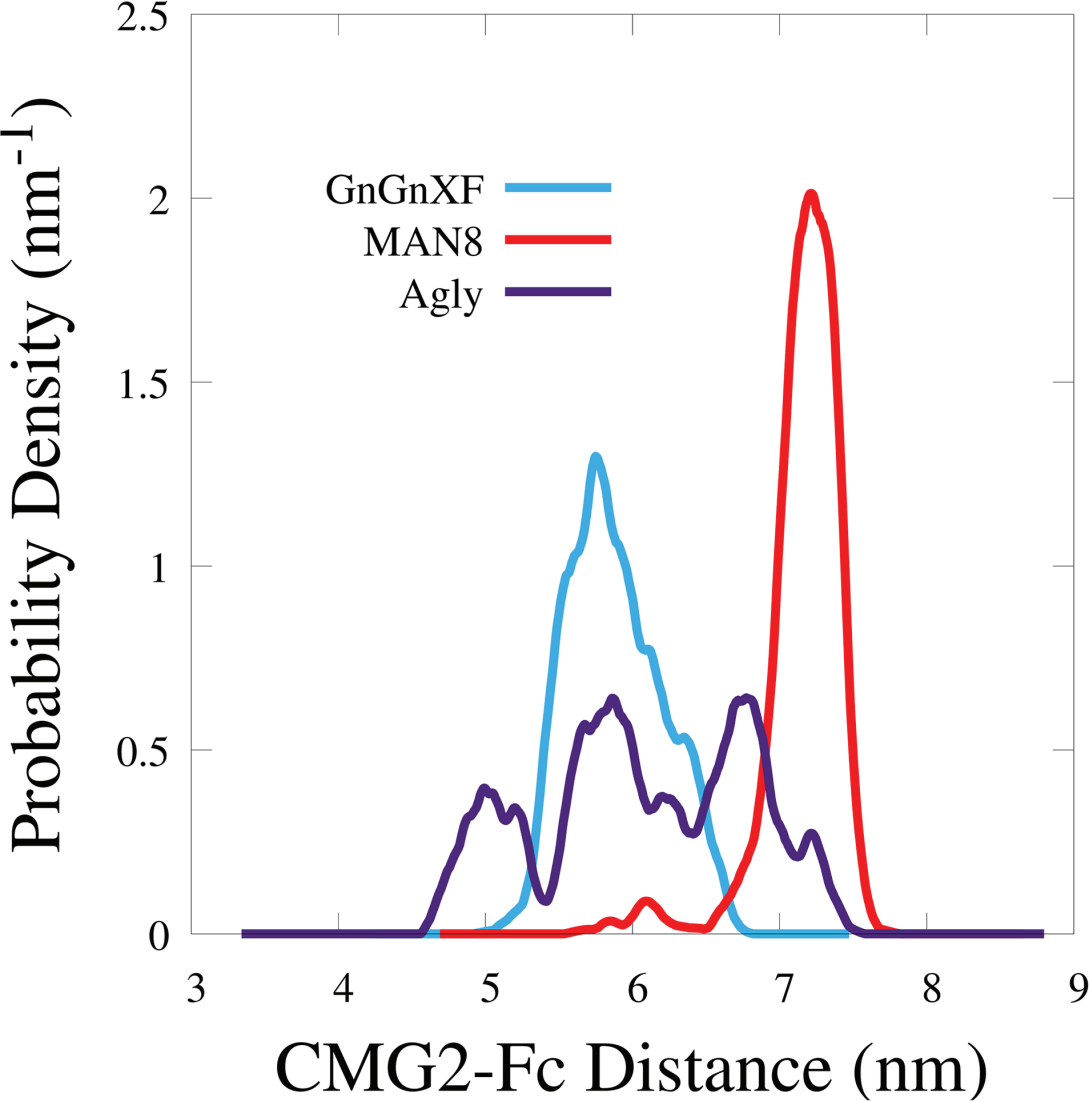
Smoothed probability density functions of the center of mass distance between the CMG2 and Fc domains for all three glycoforms. Data were smoothed with an averaging window of 0.2 nm.

To quantitatively characterize the macrostructural differences between the GnGnXF, MAN8, and Agly glycoforms, we report the center of mass (COM) distance between the CMG2 and Fc domains in Figure 9. Among the glycoforms, MAN8 has the highest COM distance with the narrowest spread at around 7.2 nm, GnGnXF and Agly have lower COM distances with wider spreads, roughly centered around 6 nm, with the Agly COM spread being the widest The significantly higher COM of MAN8 than GnGnXF and Agly is visually consistent with the final conformations in Figure 8. The large spread in Agly rCMG2-Fc COM distance is largely due to refolding of the flexible residues in and near the LNK region. The COM distance temporal profile in Supplementary Figure S10 displays a gradual increase in Agly COM distance, which tapers off around 80 ns. Thus, the larger spread in Agly COM is due to conformational changes during the simulation, not a single conformation with increased flexibility. The root mean square fluctuation (RMSF) data in Supplementary Figure S11 show increased Agly RMSF in and around the LNK region during the first 50 ns and a reduction in Agly RMSF during the latter 50 ns, which is consistent with the trending exhibited in the Agly COM distance.

### Backbone RMSD of Ordered Domains

**Figure 10:**
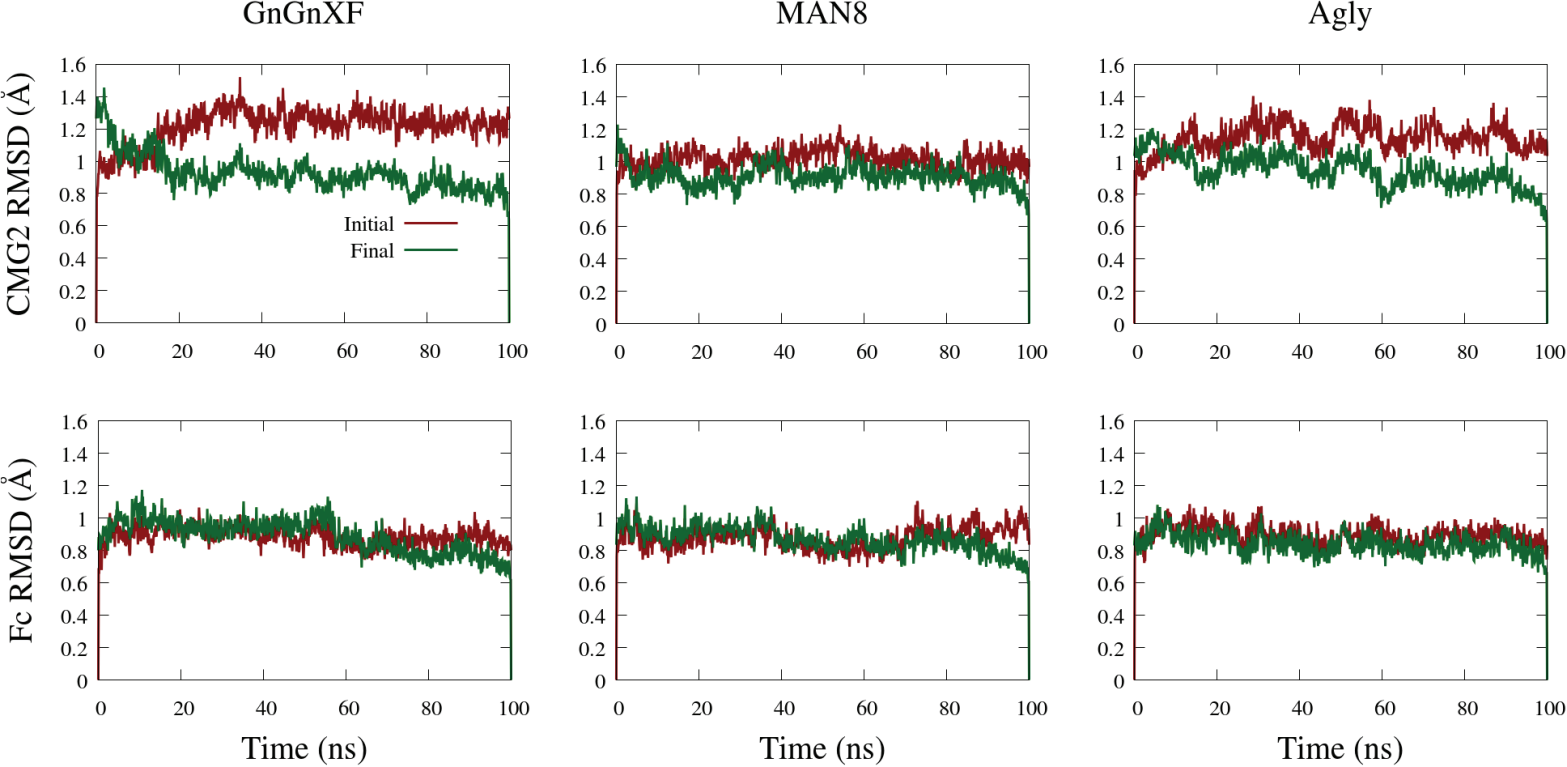
Backbone RMSD of ordered domains referenced from the initial or final conformations of the CMG2 (top) and Fc (bottom) regions of the GnGnXF, MAN8, and Agly glycoforms.

The backbone root mean square deviation (RMSD) of ordered domains referenced from the initial and final conformations of the CMG2 and Fc regions for all three simulated glycoforms is shown in Figure 10. A low RMSD indicates protein folding transition is minor and the protein structure is stable. For each monomer, the ordered domain of the CMG2 region were defined as residues 10–181, while the ordered domains of the Fc region were defined as the CH2 and CH3 regions: residues 210–308 and 315–414, respectively. Each domain’s RMSD was fit and referenced independently, and subsequently averaged to produce the plots in Figure 10. Two RMSD profiles were averaged to generate the CMG2 RMSD, and four RMSDs were averaged to generate the Fc RMSD for each glycoform.

All RMSDs are below 1.6 Å, indicating a generally conserved fold for all the ordered domains of each glycoform. This is further confirmed by the secondary structure data, provided in Supplementary Figure S12. In general, the RMSD from the final structure is lower than that of the initial, indicating conformational convergence is progressing throughout the simulations. The low RMSD and conserved secondary structure in all three simulated glycoforms indicate that there were no major refolding events; thus, the reduced activity in the TNA (Figure 5A) and the functional ELISA (Figure 7B) of the ER variant is likely not due to reduced fold stability. The Fc RMSD is noticeably lower than the CMG2 RMSD for the Agly and GnGnXF glycoforms, indicating minor fold transitions in the CMG2 domains of these glycoforms.

### Hydrophobic Solvent Accessible Surface Areas

**Figure 11:**
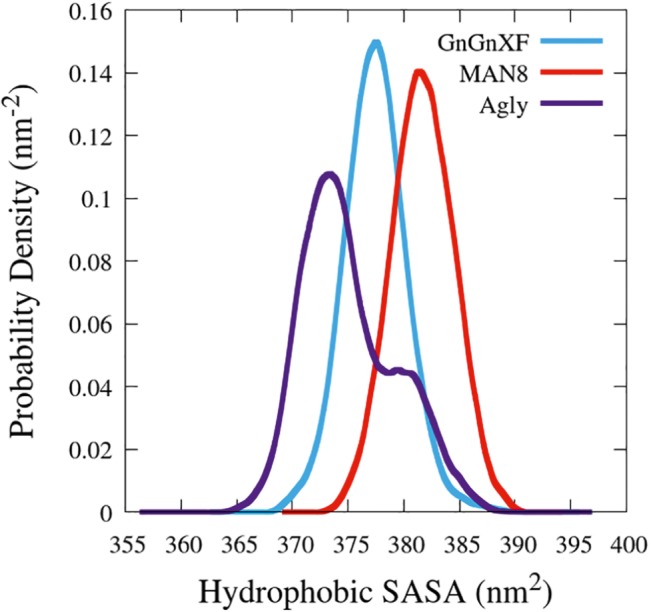
Smoothed probability density functions of the hydrophobic SASA for all three rCMG2-Fc glycoforms. Data were smoothed with an averaging window of 3 nm^2^.

The hydrophobic solvent accessible surface area distributions are reported in Figure 11. Hydrophobic SASA is defined as SASA that is associated with hydrophobic amino acid residues (ALA, GLY, ILE, LEU, MET, PHE, PRO, and VAL). The MAN8 glycoform has the highest amount of hydrophobic SASA in the simulation, while the aglycosylated has the lowest. Increased hydrophobic SASA likely yields a greater aggregation propensity (Fink, 1998).

**Figure 12:**
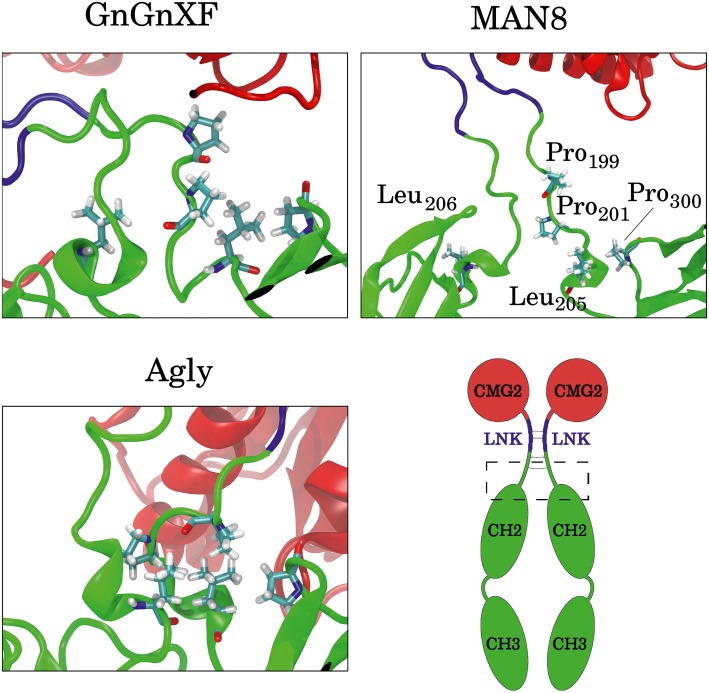
Images of the final conformations of the top five rCMG2-Fc residues that contribute to the largest positive differences in time-averaged, per-residue hydrophobic SASA of MAN8 minus Agly. The general location of these residues in rCMG2-Fc is depicted by the dashed rectangle in the schematic (disulfide bonds are indicated with solid gray lines).

To elucidate which residues contribute most to the hydrophobic SASA difference between MAN8 and Agly, the average per-residue hydrophobic SASA was calculated, and the five residues with the greatest positive difference of MAN8 minus Agly hydrophobic SASA were obtained. These residues were Pro_199_, Pro_201_, Leu_205_, and Pro_300_ of one monomer, and Leu_206_ of the other monomer. All of these residues are located in close proximity to the N-terminal region of the Fc domain, shown in Figure 12. We see that these residues are highly spread out in the MAN8 structure, moderately spread out in the GnGnXF structure, and closely associating in the Agly structure. The increased SASA in the MAN8 glycoform is consistent with the existence of the high molecular weight band in the ER variant at ~250 kDa in the SDS-PAGE results (Figure 2B).

## Discussion

In this paper, experimental and computational techniques were employed to study the effects of N-glycosylation on the expression, structure, function, and stability for anthrax decoy protein rCMG2-Fc.

### rCMG2-Fc Expression

N-glycosylation was found to strongly stabilize rCMG2-Fc *in planta* as both APO and ER variants have over two-fold higher expression level than the Agly variant, shown in Figure 1. The increase in expression level of glycosylated variants could be attributed to a decreased susceptibility of the APO and ER variants to proteases, where the steric hindrance of oligosaccharides inhibits proteolytic degradation of glycosylated rCMG2-Fc. From a manufacturing standpoint, producing glycosylated rCMG2-Fc would require less than half the production capacity of the aglycosylated form. Thus, when glycosylation is not detrimental, preserving natural N-glycosylation sites can enhance protein production. Alternatively, retaining the aglycosylated protein in the ER can also help improve yield, since the ER has fewer types of proteases than the apoplast (Doran, 2006). Furthermore, unlike the apoplast, the ER has chaperone proteins to provide folding support (Doran, 2006). It is also possible to enhance a glycoprotein’s function *via* modification of the N-glycosylation profile during expression. This can be achieved using subcellular targeting and/or the co-expression of glycan-processing enzymes or addition of enzyme inhibitors in the agroinfiltration buffer. For example, high mannose Fc N-glycosylation has been shown to enhance antibody-dependent cell-mediated cytotoxicity (ADCC), which can be achieved by targeting the protein to the ER or addition of mannosidase I inhibitor to the agroinfiltration buffer or cell culture medium (Yu et al., 2012; Xiong et al., 2018; Kommineni et al., 2019). It is worth noting that N-glycosylation of Fc is not strictly required when Fc is fused to the target protein for the purpose of increasing circulation half-life (Souders et al., 2015).

SDS-PAGE and Western blot confirmed that intact rCMG2-Fc was produced, with bands near 50 kDa (Figure 2A). There is also a band around 250 kDa in the non-reducing SDS-PAGE for the ER variant (Figure 2B), which may correspond to a high molecular weight protein aggregate. The hypothesis of increased aggregation propensity of the ER variant is supported by the hydrophobic SASA predicted from our MD simulations. MAN8 was found to have significantly higher hydrophobic SASA, with the five strongest contributing residues in the N-terminal region of the Fc domain. Protein aggregation might reduce PA binding capacity, as it is evident in the functional ELISA (Figure 7), which is later discussed in the rCMG2-Fc function section.

### rCMG2-Fc Function

The ability for rCMG2-Fc variants to sequester anthrax PA and prevent cell death was assessed using a cell-based TNA. Results are shown in Figure 5, where the ER variant has a statistically higher EC_50_ value than APO and Agly variants. The possibility of FcγR dependent toxin neutralization (Verma et al., 2009; Abboud et al., 2010; Ngundi et al., 2010) was ruled out as the EC_50_ values did not change upon FcγR blocking. This is likely because previous studies used antibodies or serum against PA that bind to PA but not necessarily blocks the its binding site to the anthrax cellular receptor CMG2. Thus, when incubated with cells, the antibody-bound PA can still bind to CMG2 and form prepore, resulting in LF and EF endocytosis. In this situation, Fc and FcγR could form an immune complex that is then sorted and degraded in the lysosome (Abboud et al., 2010). In our experiments, rCMG2-Fc competitively inhibits binding between cellular receptor CMG2 and PA. This completely eliminates LF and EF internalization.

The binding kinetics between rCMG2-Fc variants and PA were determined by BLI, results shown in Figure 6. The 37*°*C *K_D_* values were slightly lower than the 25*°*C *K_D_* values, but no appreciable difference in binding kinetics as a function of glycosylation was observed at 25 or 37*°*C. Considering the CMG2 domain is linked to Fc through a flexible linker, it is not surprising that the glycosylation of the Fc domain has minimal impact on the binding kinetics of the CMG2 domain with PA. Moreover, the sub-nanomolar affinity reported in this work is consistent with previous work on rCMG2 and PA binding kinetics (Wigelsworth et al., 2004), which is direct evidence that neither the fused Fc domain nor its glycosylation interferes with CMG2/PA binding kinetics. Even though the kinetics of all three variants were unaffected by glycosylation, it is possible that the fraction of functional protein changes over time. The BLI experiments only characterized the interaction kinetics, which are independent of the fraction of functional rCMG2-Fc on the sensor tip.

The hypothesis that the fraction of functional protein at 37*°*C is glycosylation-dependent was confirmed with the functional rCMG2-Fc ELISA, where the ER variant lost the most activity overnight, consistent with the ER variant having the highest EC_50_. However, the MD simulation data exhibit high fold stability in all glycoforms, according to the RMSD of ordered domains (Figure 10) as well as the secondary structure (Supplementary Figure S12). Thus, we hypothesize that the reduction in activity is not due reduced fold stability. Moreover, the MAN8 had the highest hydrophobic SASA among the three simulated glycoforms, indicating a higher aggregation propensity. The residues that contributed the most to the decreased hydrophobic SASA in the simulated Agly from the MAN8 were located in the N-terminal region of the Fc domain, just after the C-terminal region of the linker (Figure 12). This region is also more extended in the MAN8 glycoform, as shown in the final conformation (Figure 8) and the COM distance distribution (Figure 9). This exposure of hydrophobic residues in the thinly extended N-terminal region of the Fc domain could facilitate the aggregation with other rCMG2-Fc or protein fragments. This could explain the reduced activity for ER variant in the functional ELISA (Figure 7) and the 250 kDa band observed in the nonreducing SDS-PAGE gel for the ER variant (Figure 2B). Fusion protein aggregation has been observed in the literature for another Fc fusion protein ALK1-Fc, where a high abundance of MAN5 glycoform was found as high molecular weight aggregates (Strand et al., 2013). It is worth noting that a recent study on high-mannose type IgG showed a decrease in protection factors of backbone amide nitrogen in the CH2 domain (Fang et al., 2016), which could also contribute to the reduced activity of the ER variant. Meanwhile, Lu et al., found that high-mannose glycans have no detrimental effect on antibody stability and aggregate rate (Lu et al., 2012). The antibodies used in their study were IgG1 and IgG2, which has a molecular weight ~150 kDa. Since both the protein size and structure can affect protein stability, it is not surprising to see diverse protein stability results performed on different molecules. Within Fc-fusion proteins, structure can still vary depending on the fusion partner size and structure. However, we do expect Fc-fusion proteins with similar structure, molecular weight and conserved glycosylation site as rCMG2-Fc to likely exhibit similar behaviors.

The APO and Agly variants had no significant difference in fraction of functional protein after being incubated overnight at 37*°*C. This result is in agreement with a previous study where human IgG1s (complex N-glycan and aglycosylated) stored at 37*°*C for 21 days had no difference in aggregation or fragmentation (Hristodorov et al., 2013), suggesting the absence of glycans had no major impact on stability under physiological temperature. A previous study of rCMG2-Fc using a different linker exhibited variants with oligomannose glycan or no glycan and had similar TNA EC_50_ values for both variants (Wycoff et al., 2011), while our data indicate a significant increase in EC_50_ for the ER (oligomannose) variant over the APO and Agly variants. The linker used in Wycoff et al.’s (2011) study and this study were two serine residues followed by the upper hinge of IgG1 (SSEPKSCDKTHT) and IgG2 (SSERKCCVE), respectively. These linkers differ in both length and sequence. This study utilizes the IgG2 hinge to enhance protein dimerization due to the two additional cysteine residues for inter-hinge disulfide bond formation. Since both linker sequence and length can affect stability and function of fusion proteins (Chen et al., 2013; Lee et al., 2013), thus, it is not surprising to see a difference in toxin neutralization ability.

In this study, the expressed N-glycosylation variants were not found to functionally affect rCMG2-Fc/PA binding. However, protein expression, integrity and thermostability were affected by glycosylation. The APO variant showed the best overall performance with a high expression level, high protein integrity and thermostability. However, the debate on plant complex-type N-glycosylation is ongoing. It has been shown that plant complex N-glycans are immunogenic by the detection of anti-plant glycoepitopes antibodies in human sera (Gomord et al., 2005), despite that no adverse effects were observed when plant-made pharmaceuticals (PMPs) with complex N-glycans were applied to patients with IgE against plant glycoforms (Ma et al., 1998; Zeitlin et al., 1998; Mari et al., 2008). In addition, the plant β1,2-xylose and α1,3-fucose moieties can potentially induce rapid clearance from circulation due to the presence of IgE against those epitopes. Similarly, variants containing mannose-type N-glycans can lead to a shorter circulation half-life compared to the Agly variant, due to the presence of mannose receptor in serum (Goetze et al., 2011). However, the shorter half-life of rCMG2-Fc variants (APO and ER) can turn into an advantage when using as a post-exposure treatment (dosing is not limited), resulting in fast blood clearance of decoy-toxin complex. For prophylaxis, the Agly variant is likely the best option, considering its longer circulation half-life than other two variants.

### Summary and Future Perspectives

Glycosylation variants of an anthrax decoy protein rCMG2-Fc were successfully produced in *Nicotiana benthamiana* plants with distinct N-glycosylation patterns. The expression levels were in the range of 148–578 mg/kg LFW. The N-glycosylation profiles, characterized by mass spectrometry, were 50% high-mannose type for the ER variant and 99% complex-type for the APO variant. The rCMG2-Fc variants were all functional with sub-nanomolar dissociation rate constants regardless of N-glycosylation pattern. The higher EC_50_ of the ER variant compared with the APO and Agly variants was likely due to the loss of activity during the 37*°*C incubation condition used in the TNA assay. The loss of activity could be explained by increased aggregation of the ER variant, consistent with the SDS-PAGE and MD simulation results. To better assess the effects of N-glycosylation on protein properties, *in vitro* enzymatic glycan modification can be employed to express more uniform glycoforms. This avoids glycan heterogeneity, allowing a more accurate comparison between experimental and MD simulation data. Moreover, other proteins, especially Fc-fusion proteins can be studied using the methodology provided in this work to assess the applicability of our findings and to optimize glycoprotein therapeutic design.

## Data Availability

All datasets generated for this study are included in the manuscript and/or the Supplementary Files.

## Author Contributions

YX conceptualized, led, designed, and performed most of the experiments and wrote and edited the manuscript. KK and AB contributed equally, designed, and performed experiments, wrote and edited the manuscript. QL perform the N-glycan analysis and wrote a part of the manuscript. VK, AD, CL, RF, KM, and SN designed experiments, reviewed data, results, and interpretations, and edited the manuscript. All authors read, revised, and approved the manuscript.

### Conflict of Interest Statement

The data analyses, results presented, outcomes of this study are personal views of independent authors (YX, KK, AB, QL, VK, AMD, CBL, RF, KAM and SN). The outcomes do not reflect any financial or commercial interest of either the University of California, Davis or iBio CDMO LLC, Bryan, TX.

The authors declare that the research was conducted in the absence of any commercial or financial relationships that could be construed as a potential conflict of interest.

## Footnotes

^1^https://proteomics.ucdavis.edu/protocols-2/

^2^www.thegpm.org/crap
