# Supplementary material for: Effects of N-Glycosylation on the Structure, Function, and Stability of a Plant-Made Fc-Fusion Anthrax Decoy Protein
Source: Front Plant Sci. 2019 Jun 28;10:768. doi: 10.3389/fpls.2019.00768 (PMC6611495; doi:10.3389/fpls.2019.00768)
Supplement: Supplementary file 1 [file Table_1.docx]

**Supplementary Information**

Xiong et al. **Effects of N-glycosylation on the structure, function, and stability of a plant-made Fc-fusion anthrax decoy protein**


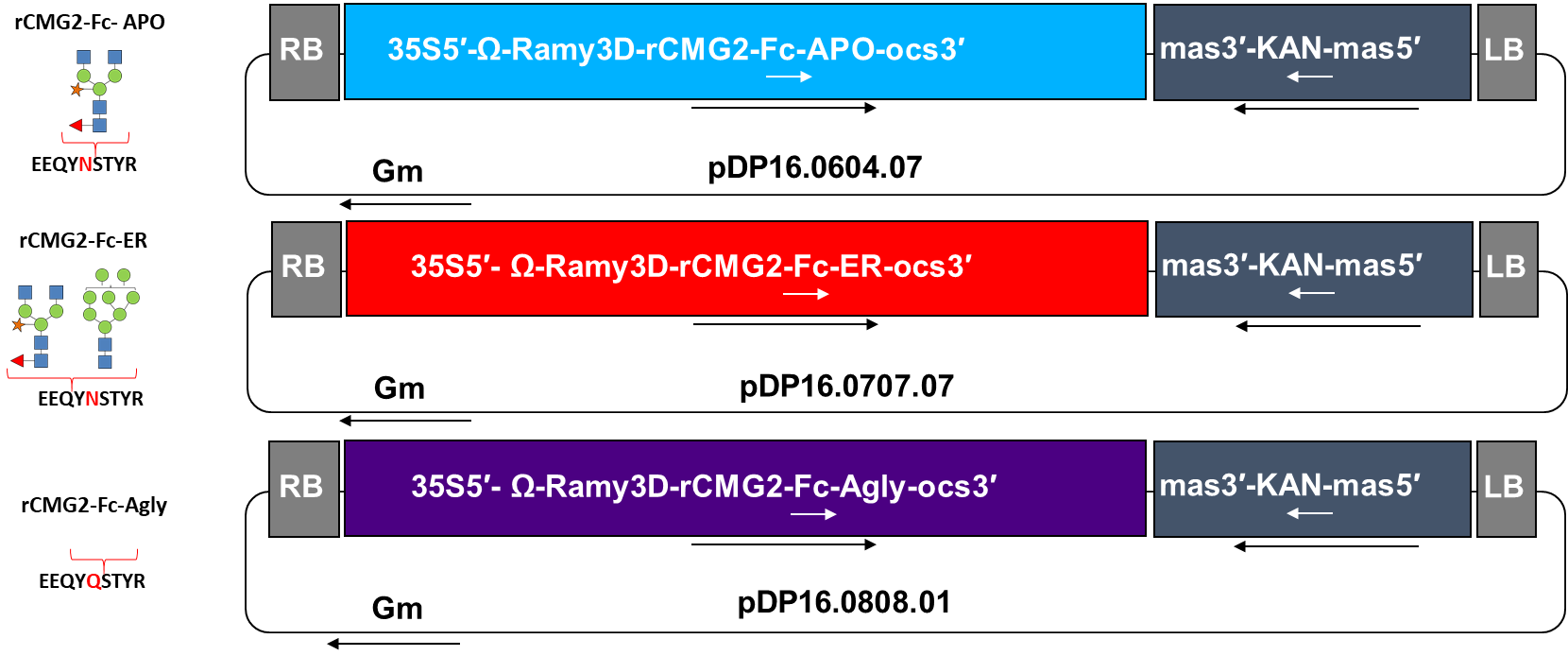


**Figure S1**. Binary vectors for rCMG2-Fc variants. RB: right border, LB: left border, 35S: Cauliflower Mosaic Virus (CaMV) promoter, Ramy3D: rice alpha-amylase 3D gene signal peptide, Ω: omega sequence, rCMG2-Fc: gene coding for the anthrax decoy fusion protein, ocs: octopine synthase terminator, mas5’ and mas3’: transcription initiation and termination sequences respectively from the mannopine synthase gene of *Agrobacterium tumefaciens*, KAN: protein encoding resistance to the antibiotic kanamycin. Predominant glycan structures are shown on the left of each vector, with N-glycosylation site written in red.

**
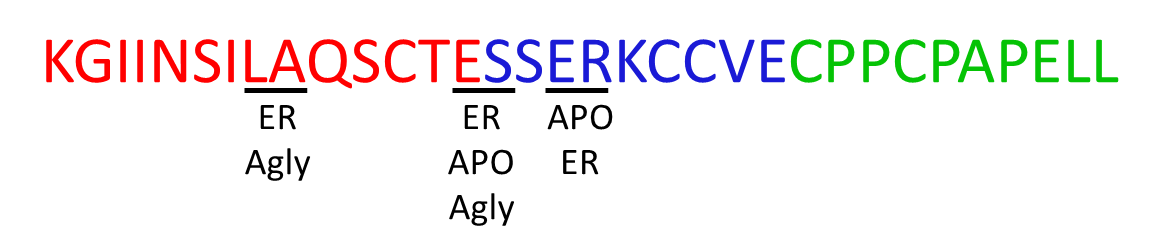
**

**Figure S2.** Proteolytic cleavage sites identified with N-terminal sequencing of the band ~25kDa in Fig. 2A. Sequence is colored coded by region: red: CMG2 C-termini, blue: linker; green: Fc N-termini.

**
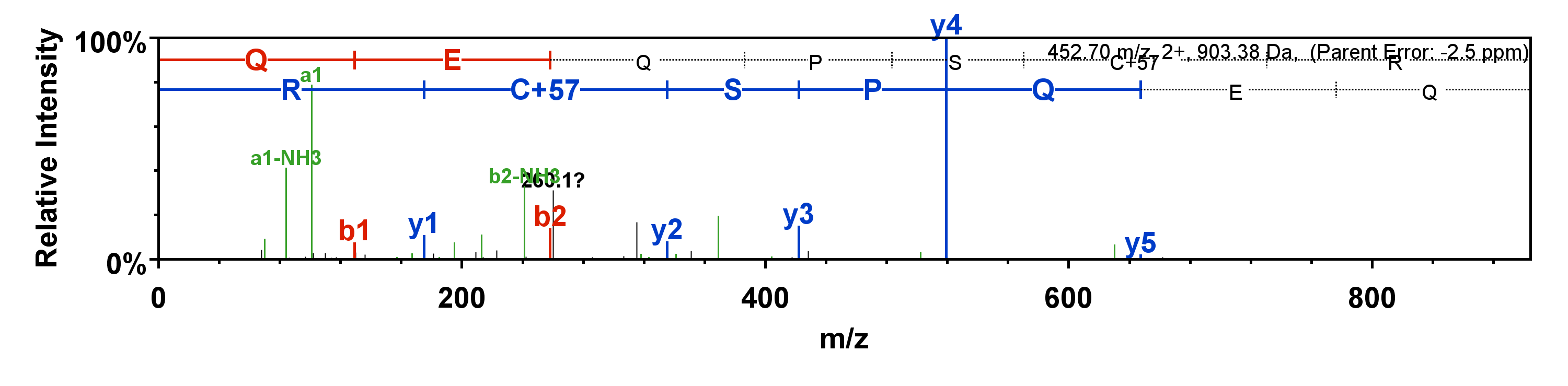
(A)**

(i)


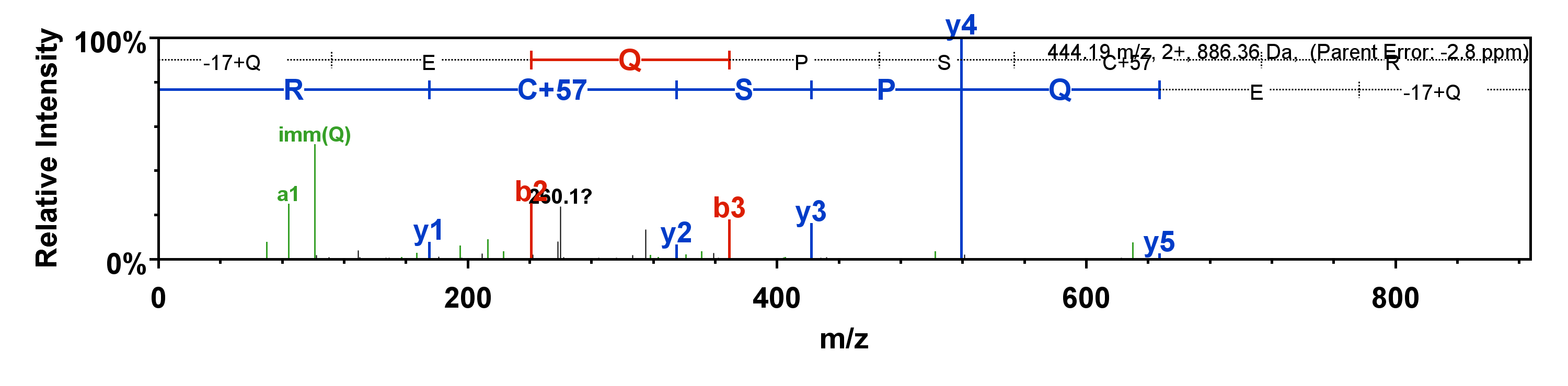


(ii)


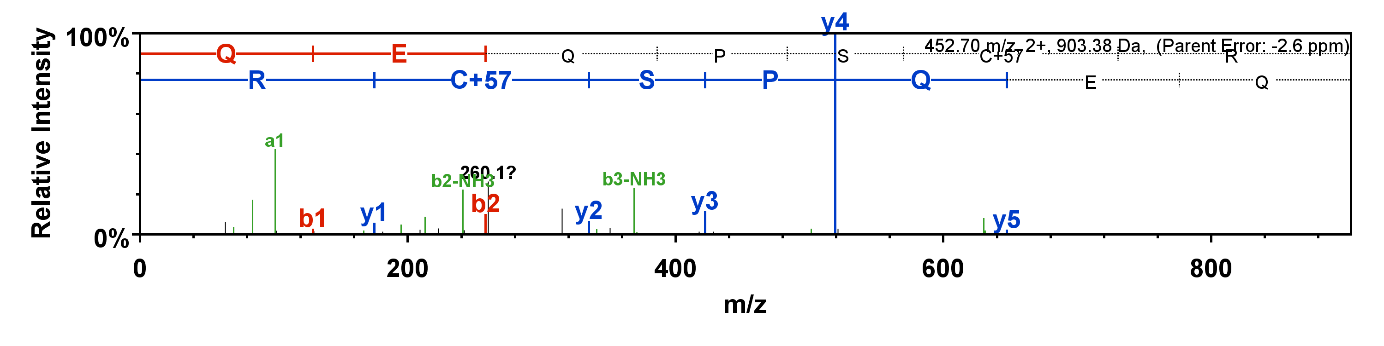


(iii)

**
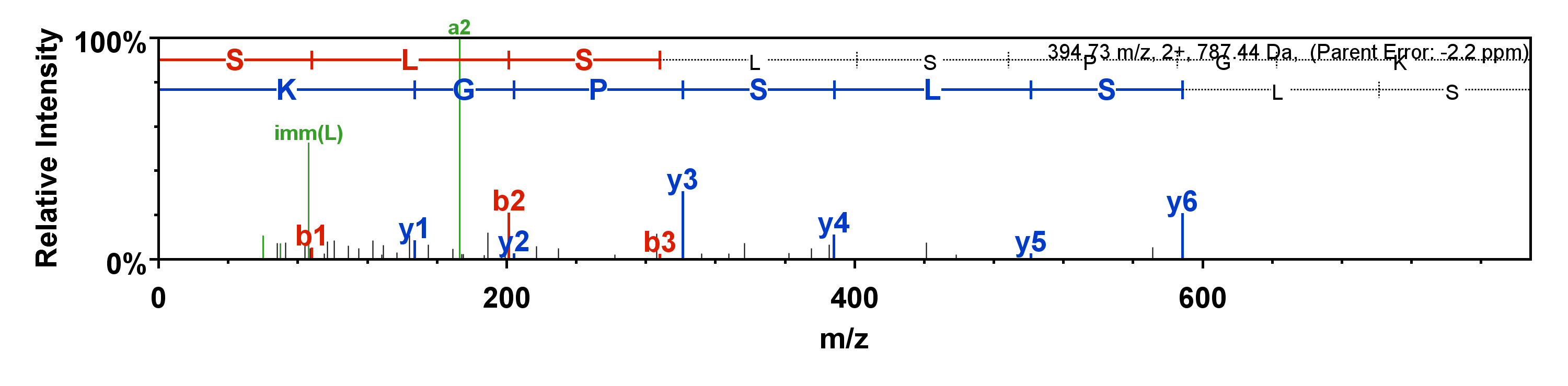
(B)**

(i)


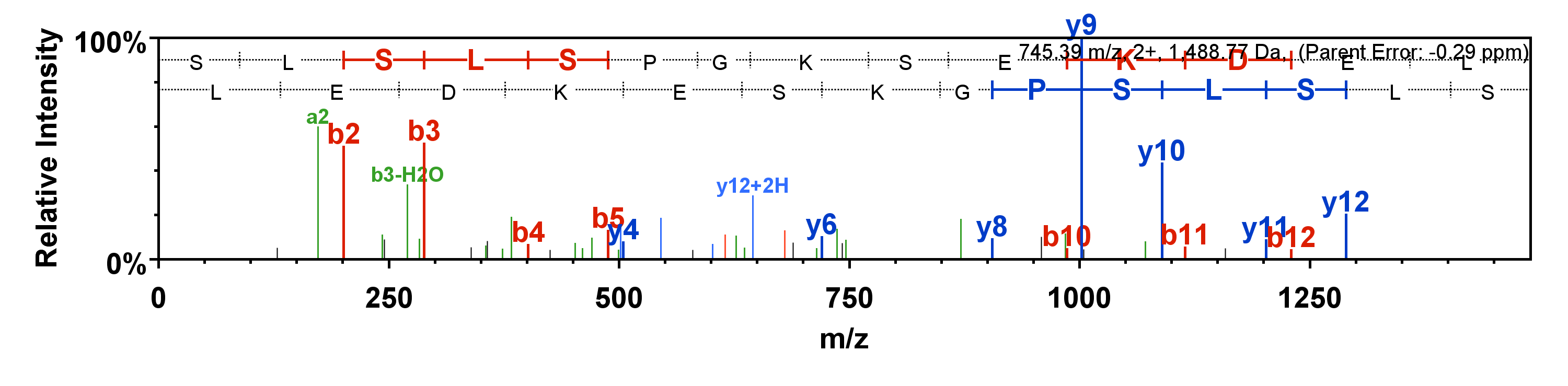


(ii)


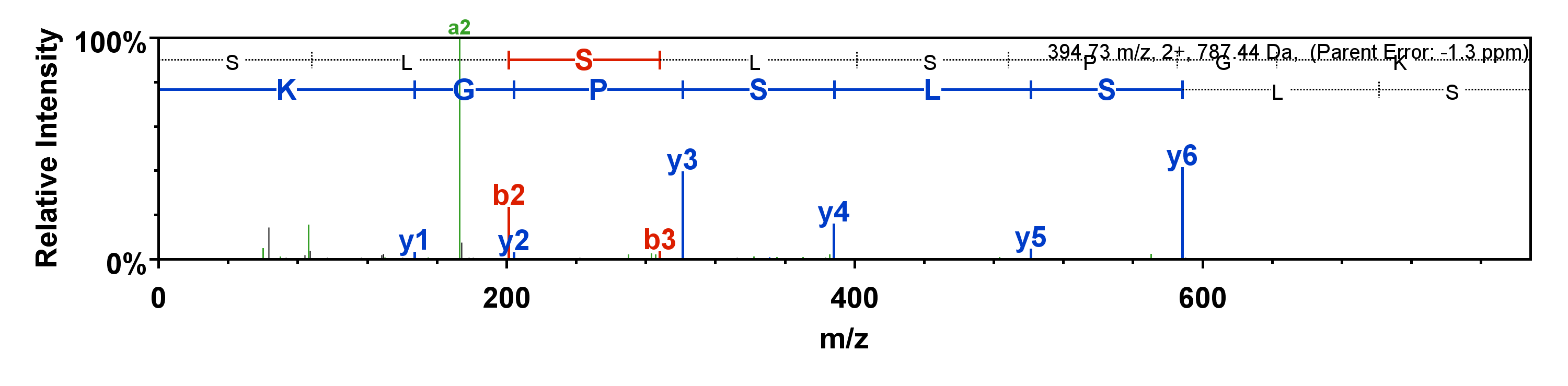
(iii)

Figure S3. Mass spectrometry analysis of rCMG2-Fc variants: (i) APO, (ii) ER and (iii) Agly. Unique peptides were identified from N-terminal (A) and C-terminal regions (B) for all three variants, confirming the protein integrity. The b ions shown on the spectrum extend from the N-terminus and y ions shown on the spectrum extend from the C-terminus of the tryptic peptides. The colors indicate the loss of ammonia or water from either b or y ions (green), doubly charged b ions (red) and doubly charged y ions (blue).

(A)
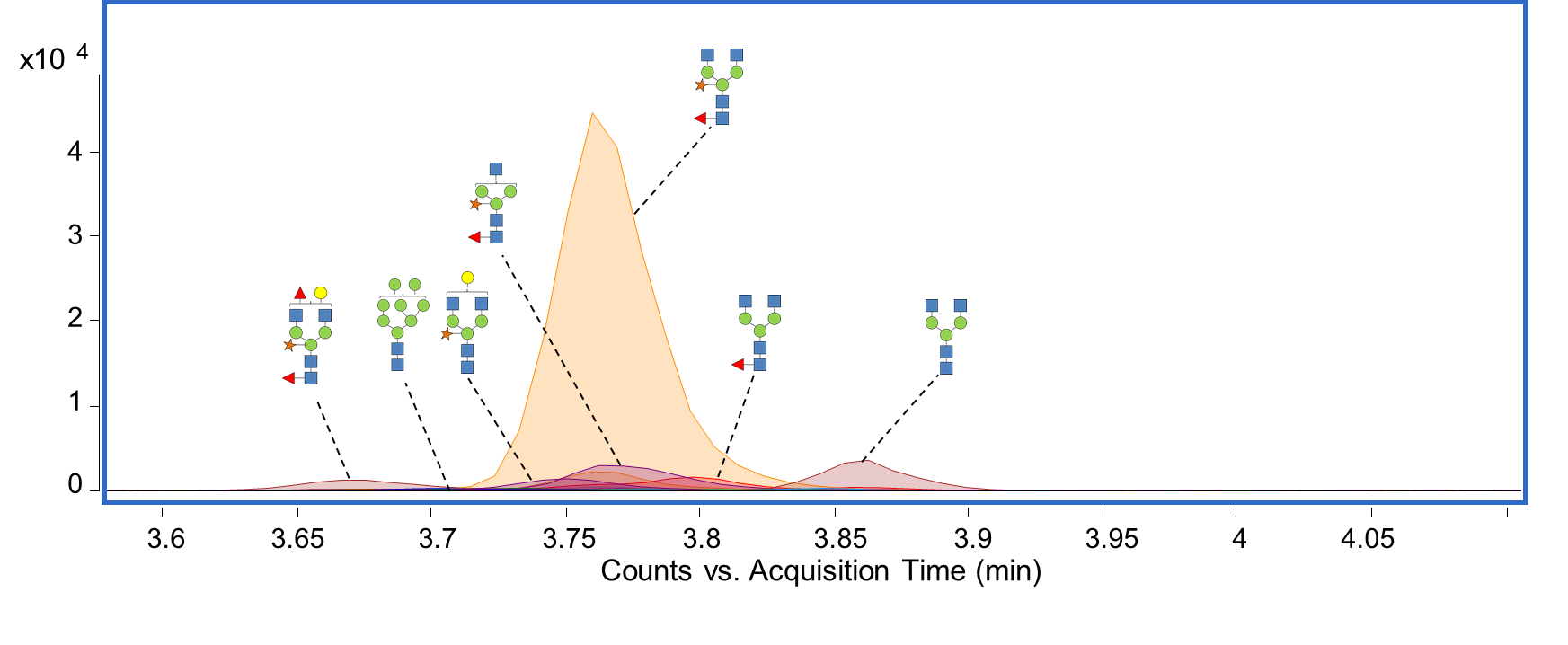


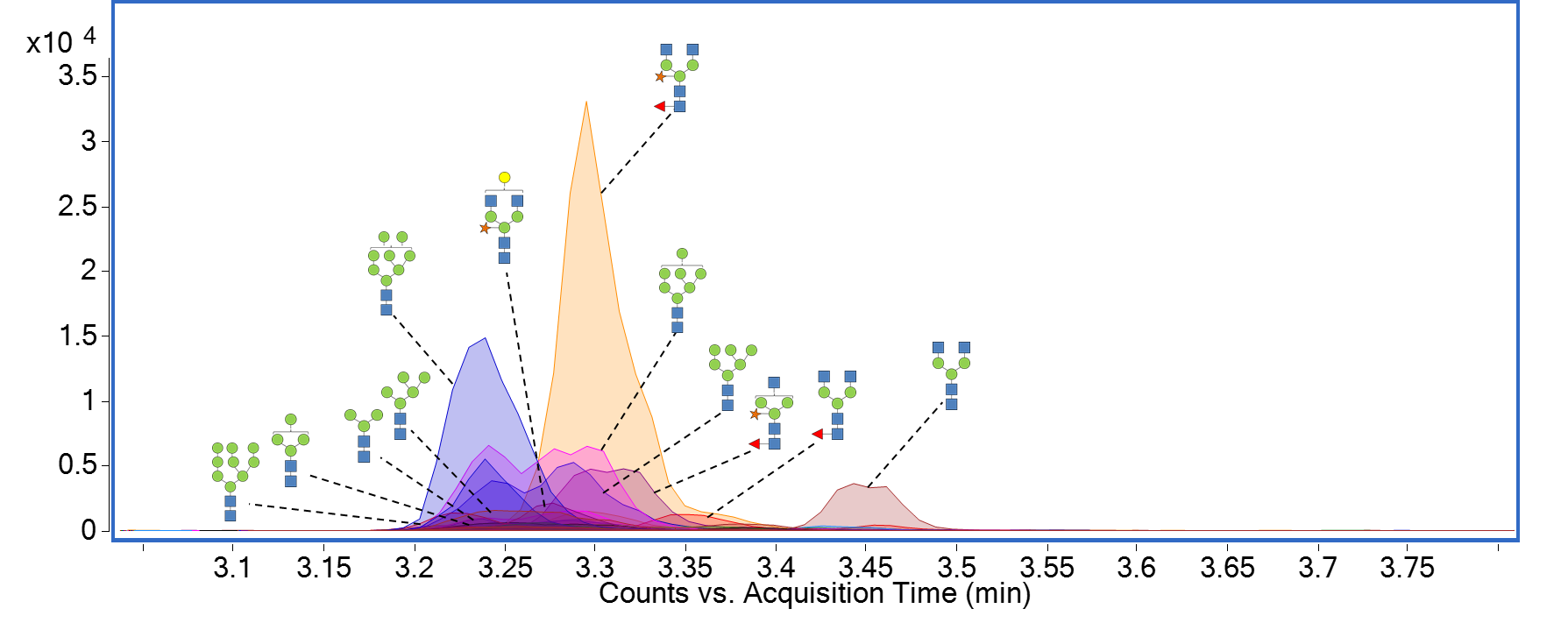
(B)

Figure S4. The Ion counts vs. Acquisition time for protein N-glycosylation quantitation from dMRM. Glycan structures are assigned to the corresponding peaks for the APO variant (A) and the ER variant (B). The relevant abundance for each structure was calculated by integrating the area under the peak.

(A)


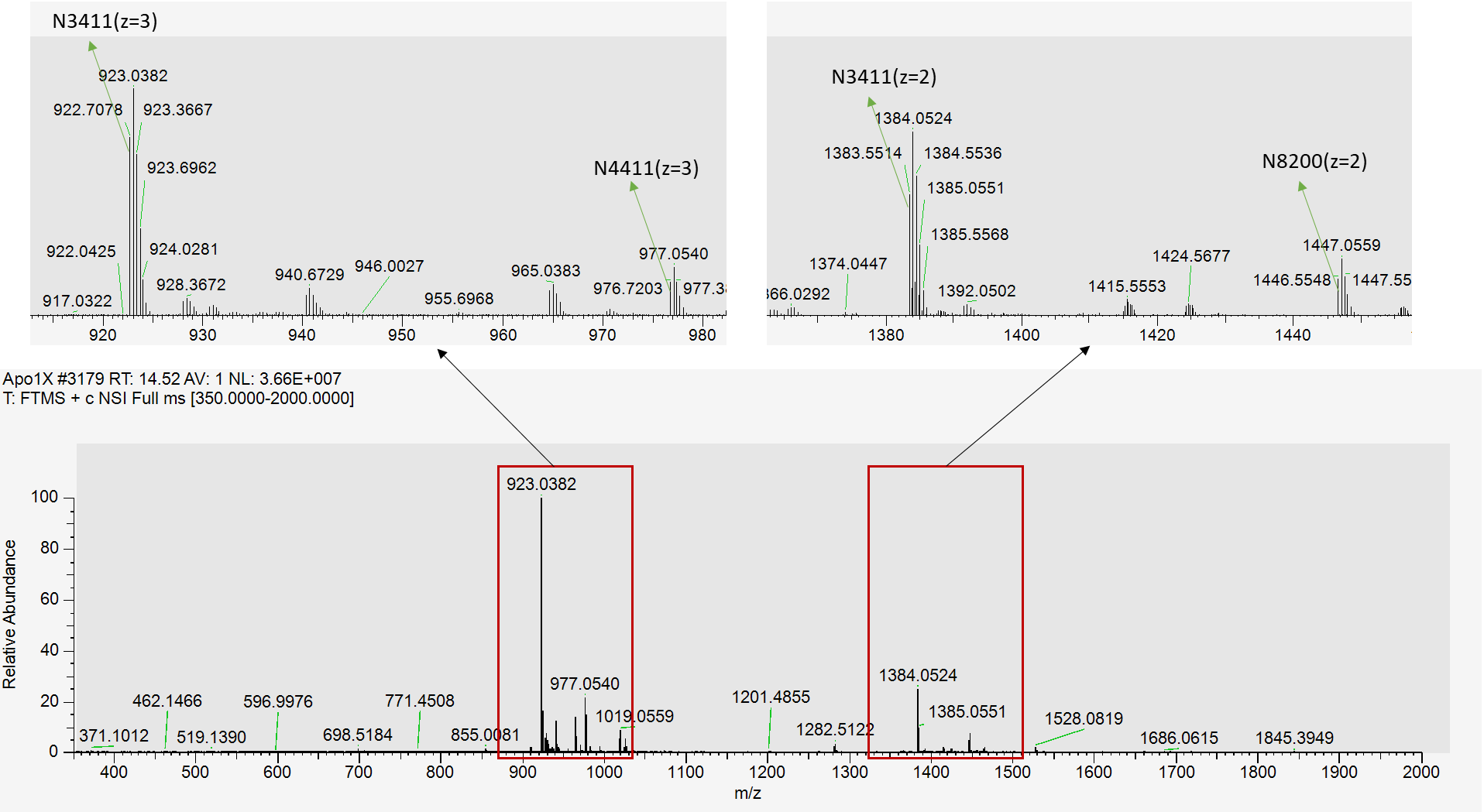


(B)

(i)


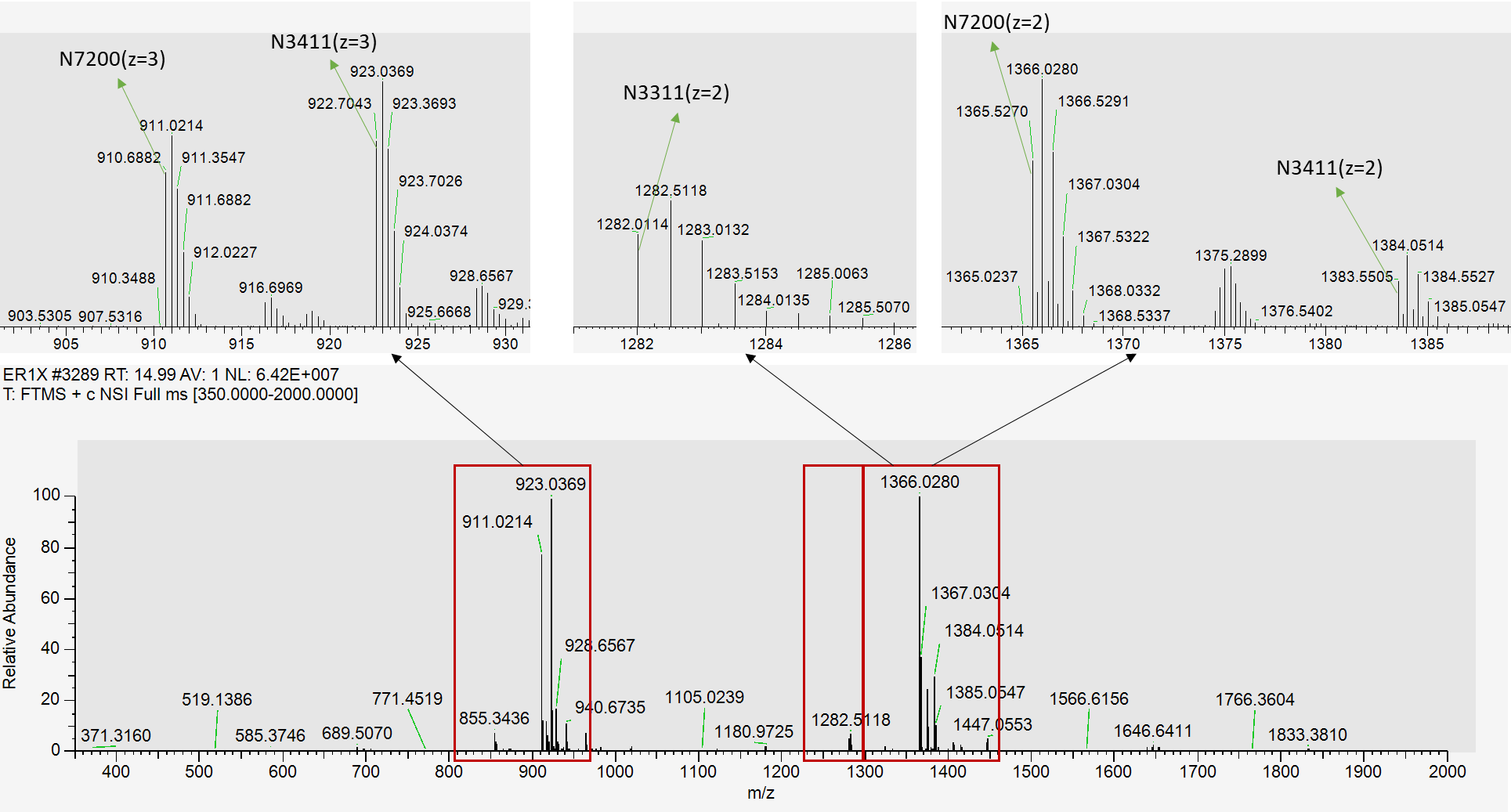


(ii)


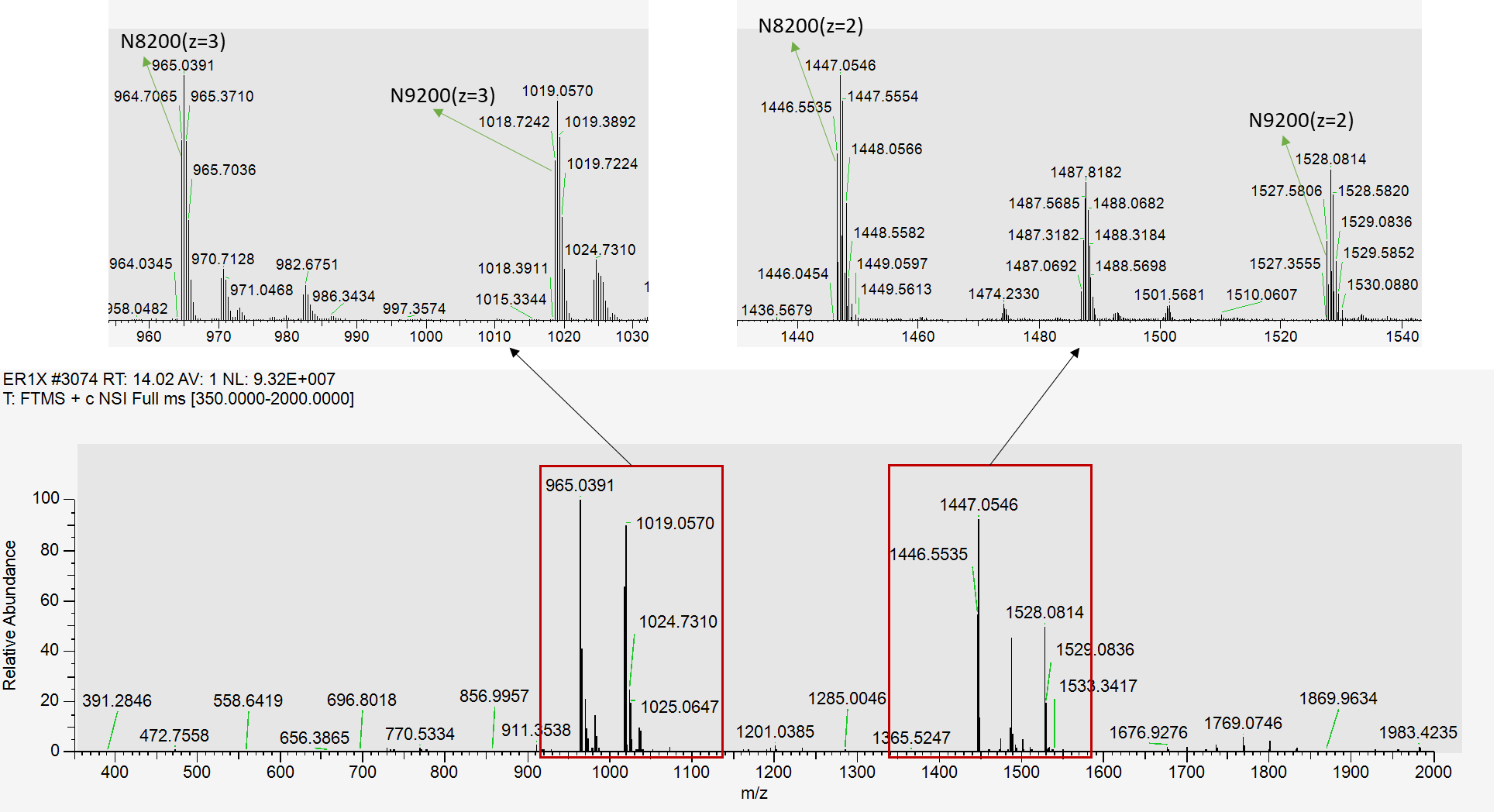


Figure S5. Representative full MS spectra for the APO (A) and ER (B) variants with glycoforms assigned to peaks. The glycans are named by the number of N-linked glycan molecules: mannose and galactose/GlcNAc/ Fucose/Xylose residues, respectively.

(A) (B)


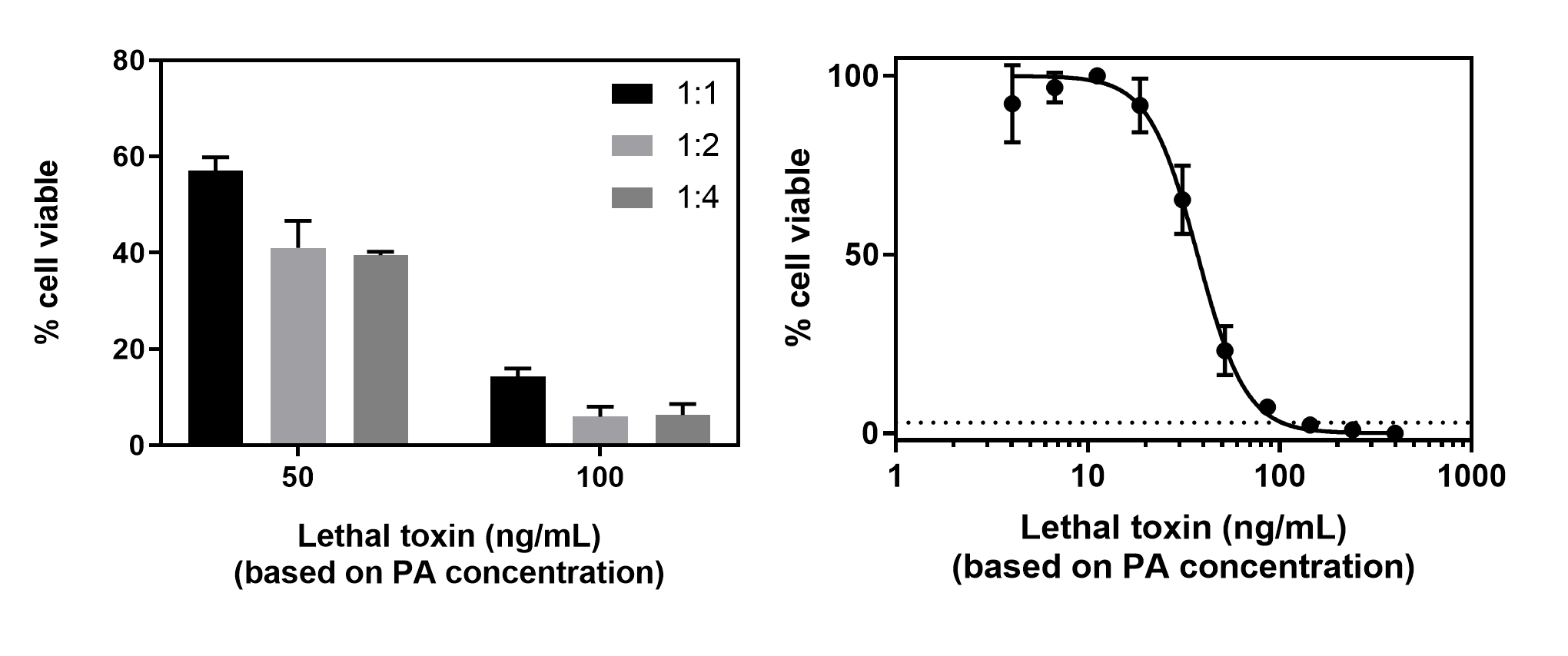


**Figure S6.** Toxin ratio and concentration optimization. Three PA to LF ratios were titrated at PA concentration of 50 and 100ng/mL (A). At both PA concentrations, increasing PA:LF ratio from 1:1 to 1:2 resulted enhanced cell killing effect, and further ratio increase has no significant impact on cell killing. 1:2 ratio was then used for LT concentration titration (B). The LT concentration at 100ng/mL (100ng/mL PA, 200ng/mL LF) was chosen for toxin neutralization assay, resulting in almost complete cell killing (97%) in absence of rCMG2-Fc.


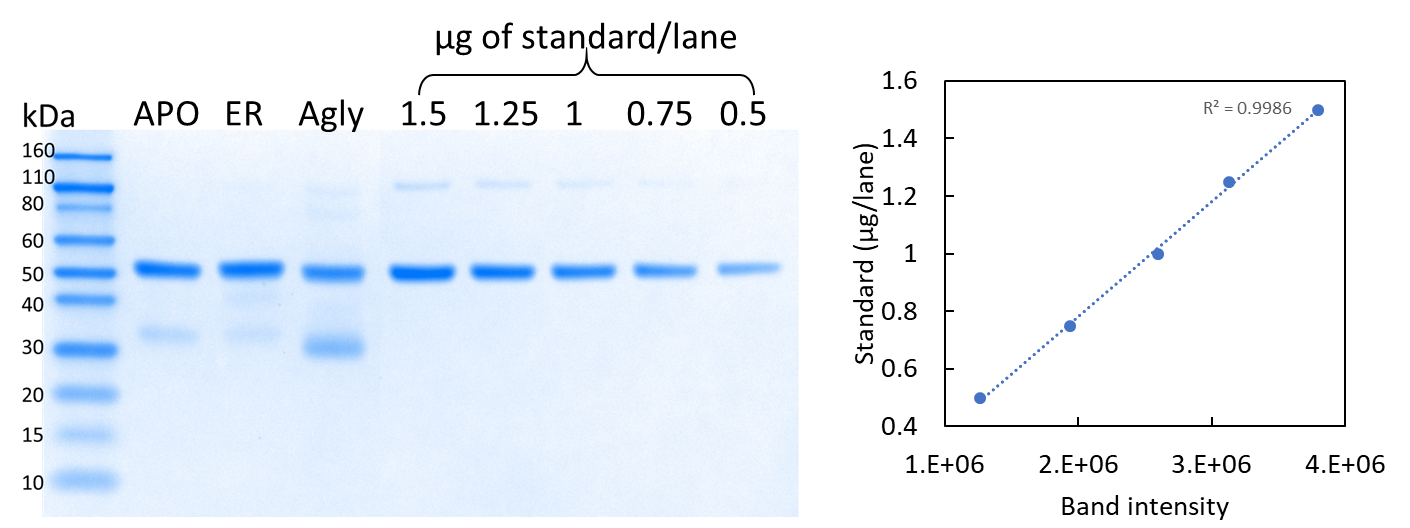


**Figure S7.** Intact rCMG2-Fc concentration determination by gel densitometry. A standard curve was established with CMG2-Fc standard purchased from Planet Biotechnology. A line was fitted to describe the relationship between the standard protein mass and band intensity, with a R^2^ of 0.9986. rCMG2-Fc variants were loaded on to the same gel as standard. The band intensity at 50 kDa was interpolated from the standard curve, and the protein concentration was determined.

(A) (B)


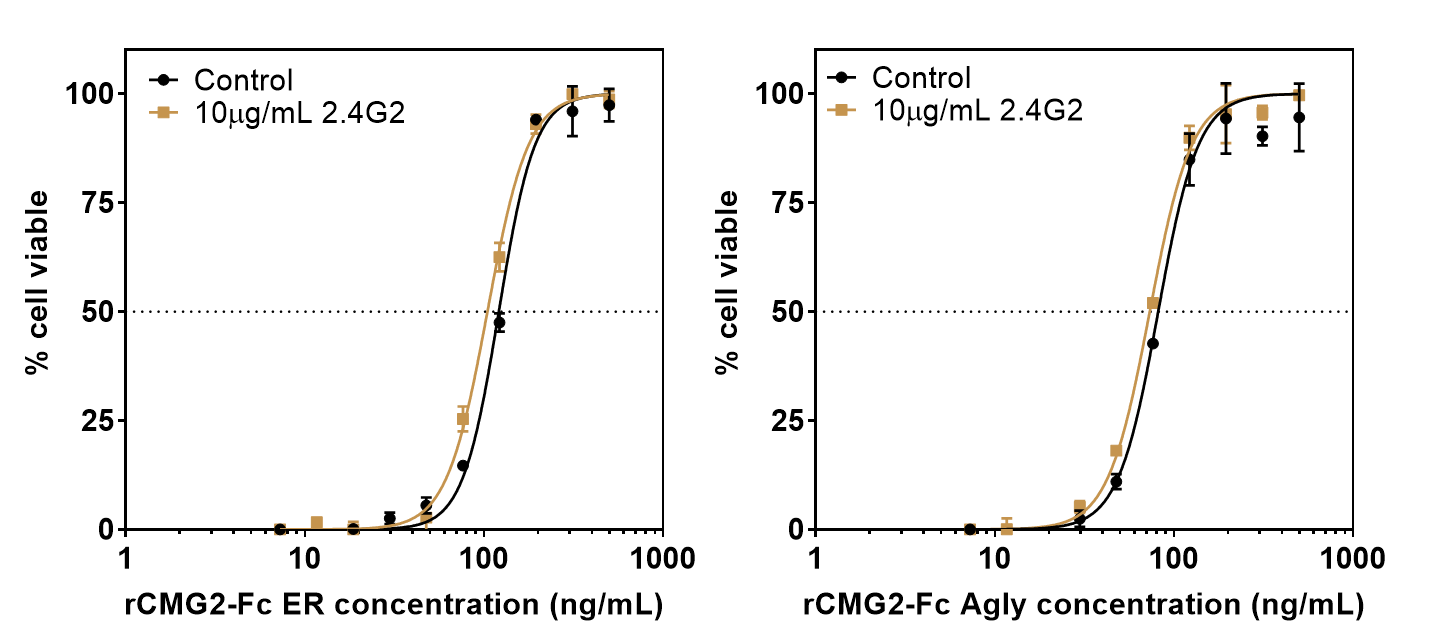


**Figure S8.** Toxin neutralization assay dose-response curves for ER variant (A) and Agly variant (B) with FcγRs blocked. Control: without anti-FcγRs antibody treatment; 10µg/mL 2.4G2: incubation of 2.4G2 (an anti-FcγRs antibody) with macrophages for 15 mins prior to TNAs.


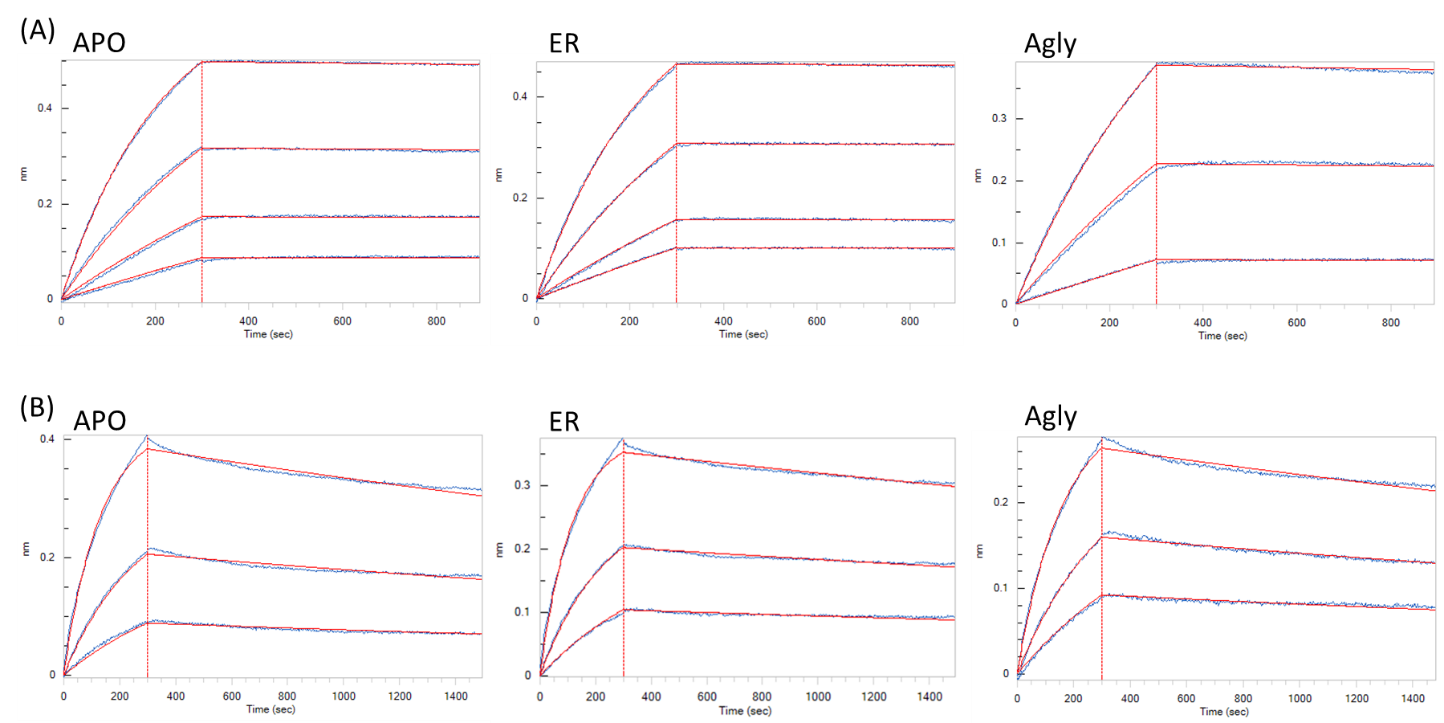


**Figure S9.** BLI sensorgrams obtained from at 25°C (A) and 37°C (B). Blue curves are BLI sensorgrams, and red solid curves are the 1:1 Langmuir fitting curves. The horizontal red dotted line separates the association phase (left) and the dissociation phase (right). The R^2^ values for all fittings are greater than 0.997.


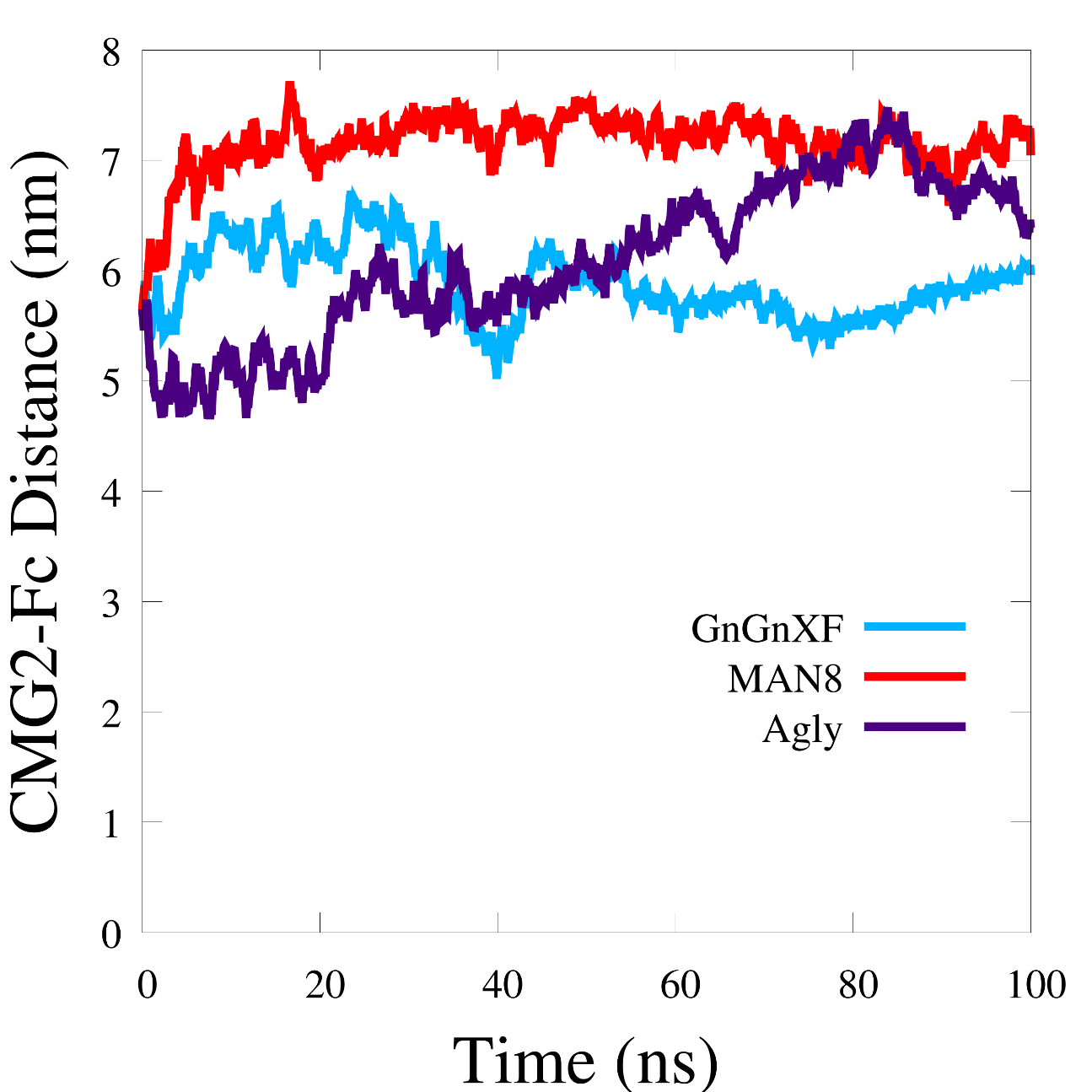


**Figure S10.** Center of mass distance between the CMG2 and Fc domains vs simulation time for the three simulated glycoforms.


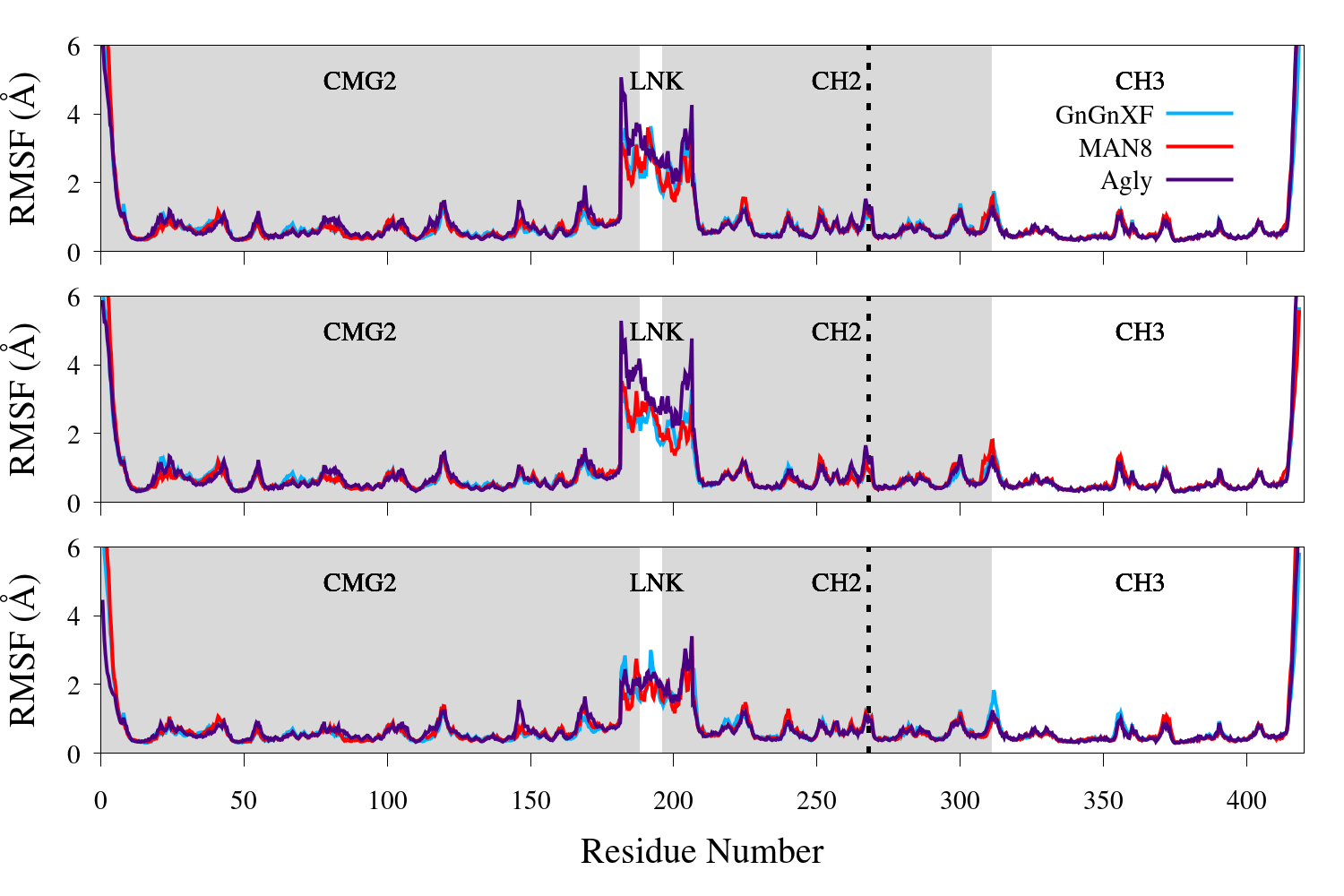


**Figure S11.** Backbone RMSF vs. residue number for all simulated glycoforms. 0-100 ns (top), 0-50 ns (middle), and 50-100 ns (bottom). The vertically dashed line occurs at Asn 268, denoting the glycosylation residue. The individual regions of CMG2, linker (LNK), and Fc CH2 and CH3 are shaded gray or white. Each domain is independently fit to remove macrostructural fluctuations.


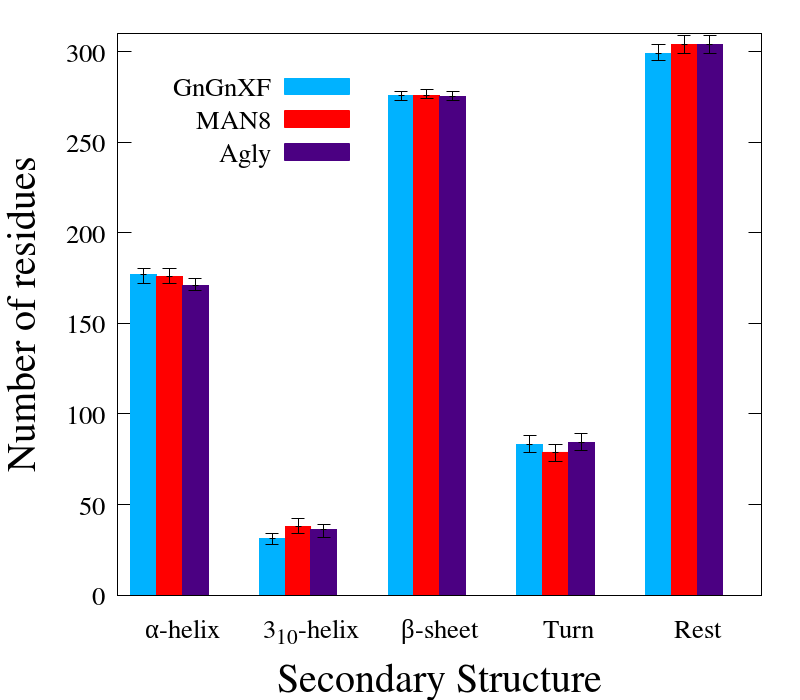


**Figure S12.** Secondary structure of simulated rCMG2-Fc glycoforms. The colored bars are median values obtained from time-varying secondary structure. The error bars are the associated first and third quartiles.
